# Guidance for DNA methylation studies: statistical insights from the Illumina EPIC array

**DOI:** 10.1186/s12864-019-5761-7

**Published:** 2019-05-14

**Authors:** Georgina Mansell, Tyler J. Gorrie-Stone, Yanchun Bao, Meena Kumari, Leonard S. Schalkwyk, Jonathan Mill, Eilis Hannon

**Affiliations:** 1 University of Exeter Medical School, University of Exeter, RD&E Hospital, Barrack Road, Exeter, Devon EX2 5DW UK; 2 School of Biological Sciences, University of Essex, Colchester, Essex, CO4 3SQ UK; 3 Institute for Social and Economic Research, University of Essex, Colchester, Essex, CO3 3LG UK

**Keywords:** DNA methylation, Epigenome-wide association study (EWAS), Multiple testing, Illumina EPIC array, Power

## Abstract

**Background:**

There has been a steady increase in the number of studies aiming to identify DNA methylation differences associated with complex phenotypes. Many of the challenges of epigenetic epidemiology regarding study design and interpretation have been discussed in detail, however there are analytical concerns that are outstanding and require further exploration. In this study we seek to address three analytical issues. First, we quantify the multiple testing burden and propose a standard statistical significance threshold for identifying DNA methylation sites that are associated with an outcome. Second, we establish whether linear regression, the chosen statistical tool for the majority of studies, is appropriate and whether it is biased by the underlying distribution of DNA methylation data. Finally, we assess the sample size required for adequately powered DNA methylation association studies.

**Results:**

We quantified DNA methylation in the Understanding Society cohort (*n* = 1175), a large population based study, using the Illumina EPIC array to assess the statistical properties of DNA methylation association analyses. By simulating null DNA methylation studies, we generated the distribution of *p*-values expected by chance and calculated the 5% family-wise error for EPIC array studies to be 9 × 10^− 8^. Next, we tested whether the assumptions of linear regression are violated by DNA methylation data and found that the majority of sites do not satisfy the assumption of normal residuals. Nevertheless, we found no evidence that this bias influences analyses by increasing the likelihood of affected sites to be false positives. Finally, we performed power calculations for EPIC based DNA methylation studies, demonstrating that existing studies with data on ~ 1000 samples are adequately powered to detect small differences at the majority of sites.

**Conclusion:**

We propose that a significance threshold of *P* < 9 × 10^− 8^ adequately controls the false positive rate for EPIC array DNA methylation studies. Moreover, our results indicate that linear regression is a valid statistical methodology for DNA methylation studies, despite the fact that the data do not always satisfy the assumptions of this test. These findings have implications for epidemiological-based studies of DNA methylation and provide a framework for the interpretation of findings from current and future studies.

**Electronic supplementary material:**

The online version of this article (10.1186/s12864-019-5761-7) contains supplementary material, which is available to authorized users.

## Background

There is increasing interest in the role of epigenetic processes in health and disease, with the primary focus of most epigenetic epidemiological studies being on DNA methylation (DNAm) [1]. Platforms such as the Illumina 450 K Human Methylation microarray (450 K array) and the Illumina EPIC Human Methylation microarray (EPIC array) have enabled the economical, high-throughput profiling of methylomic variation across large numbers of samples. In recent years a number of epigenome-wide association studies (EWAS), which aim to identify DNAm differences associated with environmental exposure and disease, have been reported for a range of complex phenotypes including cancer [2–4], autoimmune disorders [5–7], psychiatric illnesses [8, 9], neurodevelopmental disorders [10, 11] and dementia [12, 13].

Primarily due to the dynamic nature of the epigenome throughout development, across different cell types and in response to environmental exposures, much has previously been written regarding the specific nuances of performing an EWAS compared to a genome-wide association study (GWAS) of genetic variation [14–16]. However, this literature is mainly focused on study design and interpretation rather than specific analytical issues relating to the characteristics of the data. One concern that has merited some discussion relates to whether the distribution of DNAm data violates the assumptions of Gaussian linear regression [17, 18], the most commonly used analysis model as it allows for the inclusion of covariates relating to both biological and technical confounders. For each molecule of DNA in a single cell, DNAm is a binary entity, in that at any cytosine it is either present or absent. However, as almost all DNAm studies profile either bulk tissue - comprising multiple cell types - or a population of purified cells, the analyses are essentially measuring the proportion of cells (taking a value between 0 and 1) in a sample that are methylated at a specific genomic position [19]. While across the sites profiled on Illumina arrays DNAm has a bimodal distribution with peaks of hypomethylation (i.e. unmethylated sites) and hypermethylation (i.e. methylated sites), there is a significant subset of sites exhibiting intermediate levels of DNAm (proportion of methylated alleles = ~ 0.5). As the presence/absence of DNAm primarily distinguishes different cell types and tissues, in studies of a single tissue, which the majority of epigenetic epidemiology studies are, it is unlikely that the distribution at individual DNAm sites (the standard unit of analysis in an EWAS) will be bimodal. However, it is likely that the distributions will be variable and often non-normal, meaning that the assumption that the residuals of a linear regression fit are normally distributed may not hold. Furthermore, as DNAm levels are bounded by the limits of 0 and 1 it means that at the extreme ends of the distribution the variance is compressed. States of hypo and hypermethylation often define cell types and would not be expected vary biologically within a cell type, beyond any technical noise in the assay. This is exacerbated by the fact that the sensitivity of the microarray technology is less precise at these extremes of the distribution, and hence some measured variation is often present for these theoretically non-variable sites. This property of the data is called heteroskedasticity, defined as a relationship between the mean and variance of a dataset, and violates another assumption of linear regression. Although these concerns should be considered when it comes to deciding the statistical methodology, it is not currently known whether these violations are sufficient to bias analyses and introduce false positive or even false negative findings.

Consistent with studies of other types of genomic variation, another challenge for EWAS is how to account for the multiple testing burden in a typical analysis; for example, the Illumina EPIC array assays DNAm at base pair resolution for > 850,000 sites across the genome. Currently, a range of approaches are used to establish an appropriate significance threshold and there is no standard significance threshold as is used in GWAS. A common approach is a Bonferroni correction for the number of probes on the array [20–23] although this is often presumed to be too conservative as DNAm values at neighboring probes are known to be correlated [24], and many sites on the array are non-variable. An alternative, potentially more powerful, approach sets a permissible false discovery rate (FDR), and identifies the top associated sites that satisfy this criterion [25]. While FDR can be calculated by generating the empirical null distribution of test statistics [26], it is most commonly applied using the approach introduced by Benjamini and Hochberg [27]. This makes the assumption that under the null hypothesis the *p*-values across individual sites are uniformly distributed [28], which is not necessarily true. In EWAS it is not uncommon to see inflated test statistics [29, 30], even in the scenario of no true associations [31], indicating a skewed p-value distribution and perhaps reflecting unaccounted confounders such as differences in cellular composition, or certain environmental exposures such as smoking. This variation in the distribution of p-values across studies means that the FDR approach often demonstrates variable behaviour making it challenging to compare results across studies. A better approach would be to estimate the number of independent tests performed in a EWAS and make the appropriate adjustment to the significance level. Saffari and colleagues have previously applied the methodology successfully used for GWAS to DNAm data profiled on the Illumina 450 K array [32] in an attempt to establish a standard multiple testing threshold, however this is yet to be repeated for the EPIC array.

In this study, we used a large population based study, Understanding Society (*n* = 1175), where DNA extracted from whole blood was profiled using the EPIC array [33, 34] to investigate potential statistical biases of DNAm association analyses, with the goal of providing recommendations for future epigenetic epidemiology studies. First, we used a permutation procedure to establish an appropriate significance threshold that accounts for the multiple testing burden of the EPIC array. Second, we investigated whether the assumptions of linear regression are satisfied when measuring DNAm as beta-values and whether any violations bias the results of DNAm studies. Although transformations of beta-values (e.g. conversion to M-values [18]) have been proposed in order to better satisfy the assumptions of linear regression, these approaches have not been unanimously adopted by the community therefore we seek to determine the validity of studies that analysed beta-values. Finally, we used the significance threshold derived from our simulations to explore the statistical power of DNAm studies across various scenarios. These results of our analyses will inform the optimal approach to designing and analysing DNAm data.

## Results

### Estimating a multiple testing corrected significance threshold for the EPIC array

After a stringent quality control (QC) pipeline (see Methods) and the exclusion of DNAm sites that may be technically biased by either the presence of genetic variants or cross-hybridisation to multiple genomic loci, our final dataset included DNAm estimates for 804,826 sites across the autosomes and X chromosome derived from 1175 individuals. Applying the Bonferroni correction formula for multiple testing, the significance threshold for hypothesis testing would be set to *P* < 6.21 × 10^− 8^ (0.05/804,826). In order to establish a significance threshold for EPIC array DNAm studies that controls for the number of independent tests (as opposed to the total number of sites tested), we used a permutation approach previously applied to GWAS [35] and 450 K array DNAm studies [32]. This method preserves the correlation structure between sites and simulates null association studies by randomly assigning case control status. Repeating this process 100 times generates the distribution of *p*-values obtained by chance. From this distribution we calculated the 5% family-wise error rate (FWER) to be 9.42 × 10^− 8^ (Additional file 1: Figure S1). Using the inverse of the Bonferroni correction formula this is equivalent to correcting for 530,639 independent tests (0.05/9.42 × 10^− 8^), a reduction of 34.1% compared to the total number of sites included in the analysis.

### Estimating multiple testing corrected significance threshold for a genome-wide DNAm study

**Fig. 1:**
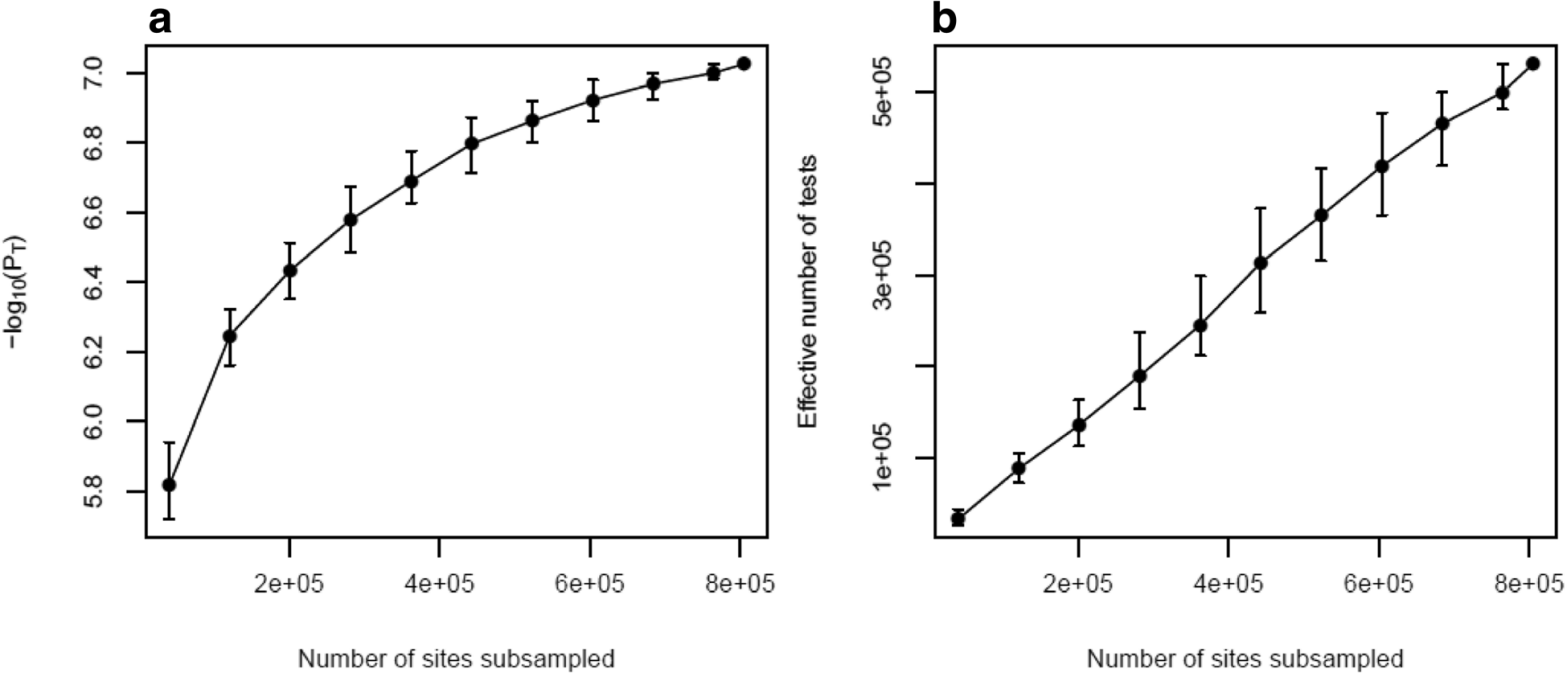
Subsampling sites on the EPIC array to estimate a genome-wide significance threshold. Line graphs depicting the relationship between the number of EPIC array DNA methylation sites (x-axis) and **a**) the 5% family-wise error rate (FWER) (−log_10_(p-values); y-axis) and **b**) the mean effective number of tests (y-axis) estimated from 1000 simulated null association studies. Error bars present the 95% confidence intervals from 1000 simulations. The final point includes all DNA methylation sites on the EPIC array and therefore could not be resampled to generate a confidence interval

**Fig. 2:**
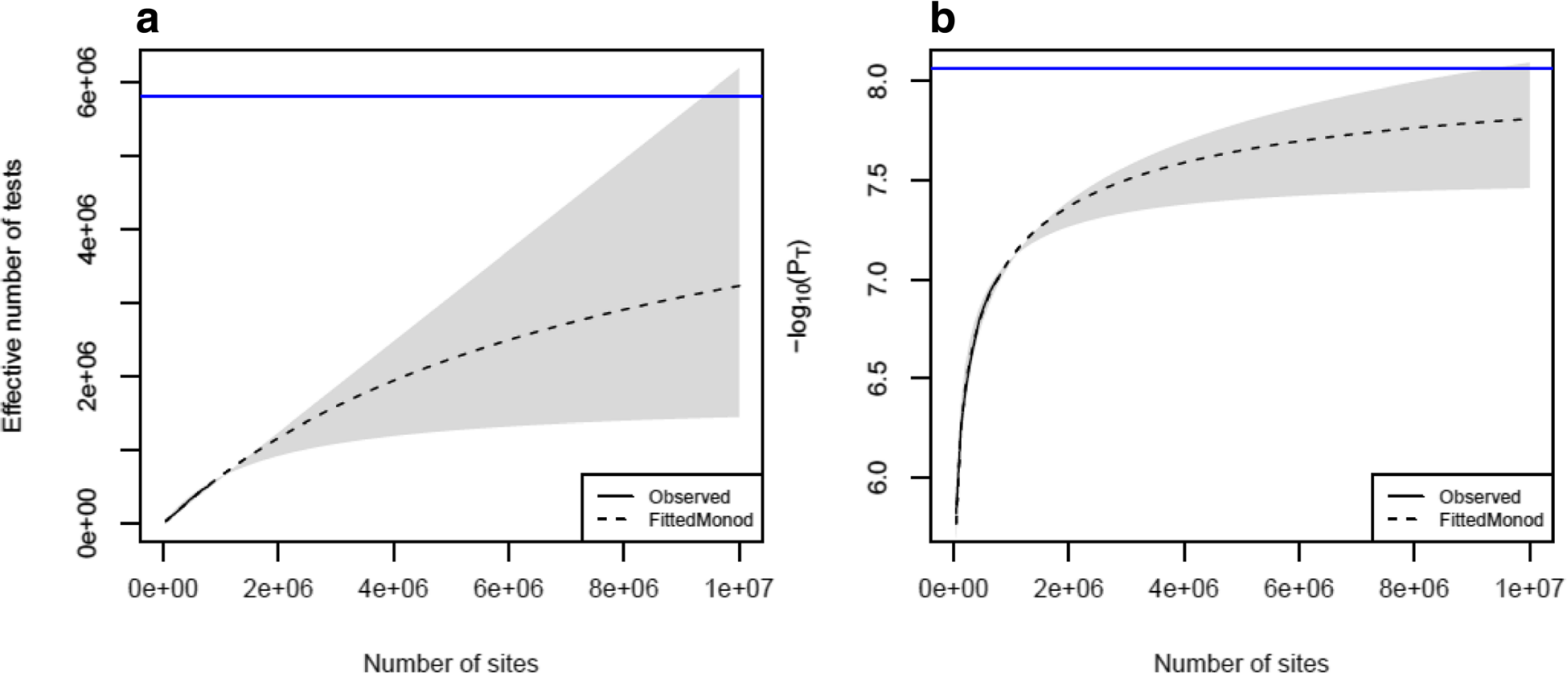
Extrapolation to a genome-wide significance threshold. Line graphs depicting the relationship between the number of DNA methylation sites (x-axis) and **a**) the effective number of independent tests (y-axis) and **b**) the multiple testing corrected threshold (−log_10_(*p*-value); y-axis) estimated after fitting a Monod function to the observed data presented in Fig. 1b. The observed values are plotted as the solid black line, and the estimated Monod model is plotted as a dashed line. The grey shaded region represents the 95% CI created by fitting a Monod model to the 95% CI of the subsampled data. The blue horizontal line represents the estimated asymptote of the Monod model of 5,803,067 independent tests equivalent to a genome-wide significance threshold of 8.62 × 10^− 9^

As DNAm microarrays only profile a subset of the ~ 28 million potentially methylated sites in the human genome, the threshold calculated above is specific to an EPIC array-based experiment and hence we will refer to it as an “experiment-wide significance threshold”. Next, we were interested in using our permutations to extrapolate from this experiment-wide threshold to a significance threshold that accounts for all variation in DNAm across the genome. Given the correlation in DNAm between proximal DNAm sites, the content of the EPIC array provides some information about neighboring sites that are not directly profiled. Continuing to increase the genomic coverage of the microarray should, therefore, have diminishing returns in terms of novel association tests as we can use the sites present on the array to infer the status of other unmeasured neighboring sites. In order to model the information gain in terms of number of independent tests as the coverage of the microarray increases, we applied our permutation procedure to subsamples of DNAm sites at increasing densities (*x*_*i*_ = 5, 15%, …, 95%). For each density, we estimated the significance threshold 100 times and calculated the mean 5% FWER (denoted P_Ti_ for density i%). These estimated P_Ti_ values are plotted in Fig. 1a, and demonstrate the expected monotonic, non-linear relationship where P_Ti_ becomes more significant as the number of sites sampled increases. Each P_Ti_ value was then used to calculate the effective number of independent tests (m_i_) at density i% using the inverse of Bonferroni formula (m_i_ = 0.05/P_Ti_). Again, we observe a monotonic relationship where the effective number of tests increases as the proportion of sites sampled increases (Fig. 1b; Additional file 2: Table S1). As the proportion of additional independent tests should decrease as the number of sites increases, this relationship is expected to be non-linear and converge to an asymptote which represents the total number of independent tests across the genome. These properties can be represented by the Monod function, which was originally proposed for the growth of microorganisms but is applicable to scenarios where subsequent growth is increasingly restricted over time. In this application, continually increasing the number of sites profiled in an experiment leads to smaller and smaller increments in the number of independent sites tested until all variation in DNAm is captured. This upper limit represents the total number of independent tests in the genome and is the value we want to estimate in order to determine the genome-wide multiple testing burden. We observe that this non-linear behaviour only starts to appear after ~ 600,000 sites. Fitting a Monod function to the subsampling results, we estimated the asymptote to be 5,803,067 (Fig. 2a) reflecting the total number of independent tests across the DNA methylome. Compared to the total number of sites in the genome, this is a reduction of 79.3%. Calculating the Bonferroni corrected significance threshold based on this estimate gives a methylome-wide significance threshold of 8.62 × 10^− 9^ (=0.05/5.80 × 10^6^) (Fig. 2b). Comparing this to a Bonferroni corrected significance threshold for all sites in the genome of 1.79 × 10^− 9^ (0.05/2.8 × 10^7^), our estimate is almost an order of magnitude smaller. The Monod function was also fitted to the subsample 95% confidence interval (CI) limits, estimating a 95% CI for the asymptote of 1.69 × 10^6^ to 3.36 × 10^13^, which equates to a 95% CI of 2.97 × 10^− 8^ to 1.49 × 10^− 15^ for the methylome-wide significance threshold.

### Testing the assumptions of linear regression for DNAm analyses

**Fig. 3:**
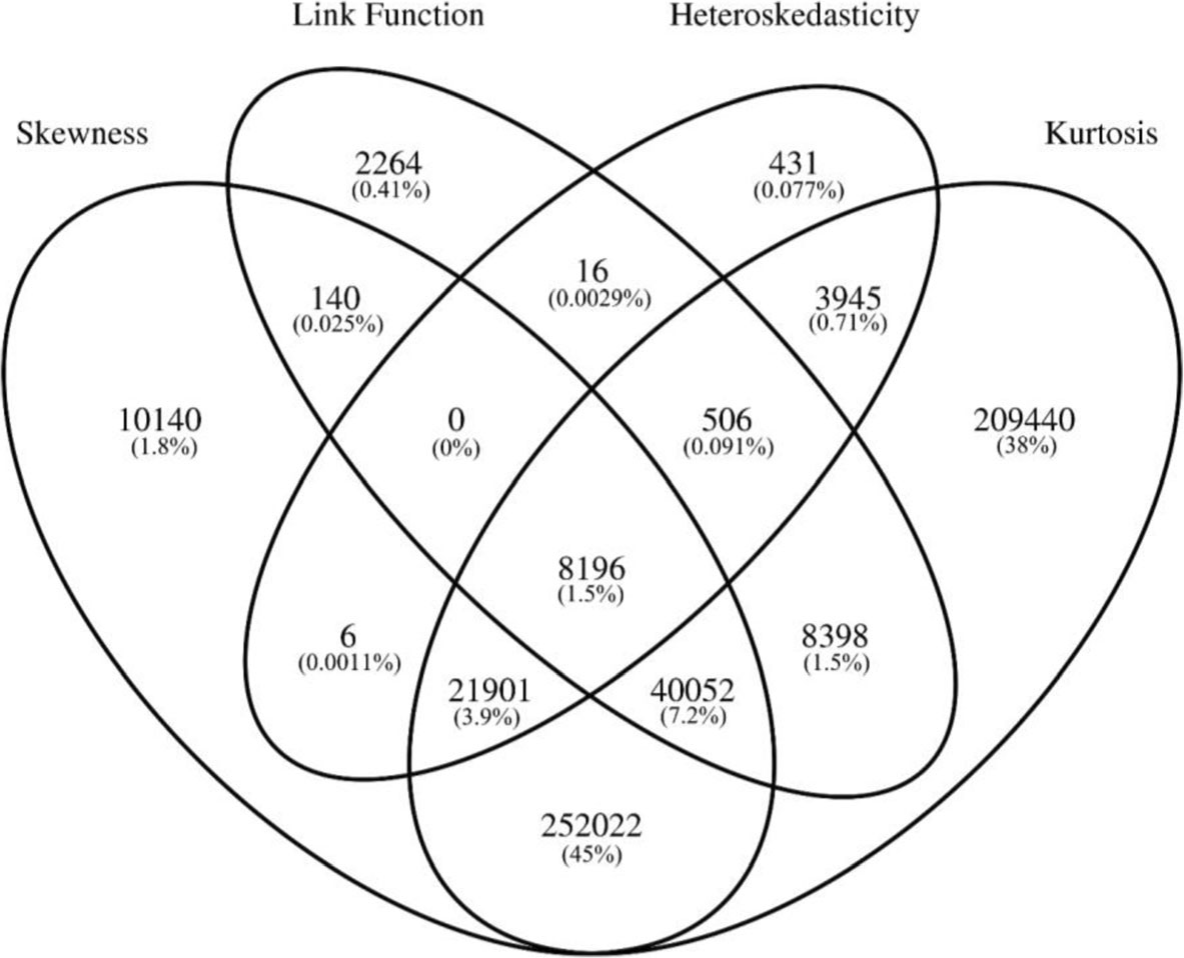
Overlap of significant violations of linear regression assumptions. Venn diagram depicting the overlap of DNA methylation sites significant for each test of a linear assumption (*P* < 9.42 × 10^− 8^). Presented are the number of overlapping DNA methylation sites along with the percentage of all tested sites shown in brackets

To assess whether the assumptions of linear regression are satisfied, we performed an EWAS of age, a trait known to robustly co-vary with DNAm at multiple loci [21, 36]. The four assumptions of linear regression were assessed using four statistical tests implemented within the *gvlma* R package [37]. Specifically, these were tests for i) skewness, an asymmetrical distribution of the residuals, ii) kurtosis, a non-bell-shaped distribution of the residuals, iii) incorrect link function, a non-linear relationship between independent and dependent variables, and iv) heteroskedasticity, inconstant variance of the residuals (Additional file 1: Figure S2). In addition, a global test was performed providing an omnibus test of the four individual statistical tests. QQ plots of all five tests demonstrated dramatic inflation of *p*-values smaller than expected by chance (Additional file 1: Figure S3), indicating that the null hypothesis that DNAm data meets the assumptions of linear regression can be rejected for a large number of DNAm sites. Based on the experiment-wide significance threshold we previously derived for the EPIC array (i.e. *P* < 9.42 × 10^− 8^), 71.8% of sites rejected the null hypothesis for at least one assumption, with the majority of sites having non-normal residuals that exhibited evidence of excess skewness (41.3%) or excess kurtosis (67.6%) (Table 1). Furthermore, the specific DNAm sites whose residuals were skewed overlapped with the sites whose residuals were kurtotic (i.e. either highly or shallowly peaked) (Fig. 3). A much smaller percentage of sites reject the null hypothesis in favour of a non-linear model (7.4%) or heteroskedasticity (4.3%).

**Table 1:** Summary of DNA methylation sites significantly rejecting the assumptions of linear regression

	Global	Skewness	Kurtosis	Link Function	Heteroskedasticity
N reject null hypothesis	577,919	332,457	544,460	59,572	35,001
% reject null hypothesis	71.8	41.3	67.6	7.4	4.3

For each of the 5 tests performed by the *gvlma* package the number and percentage of DNA methylation sites with significant *p*-values (*P* < 9.42 × 10^− 8^) are reported

### Characterising DNAm sites that infringe the assumptions of linear regression

**Fig. 4:**
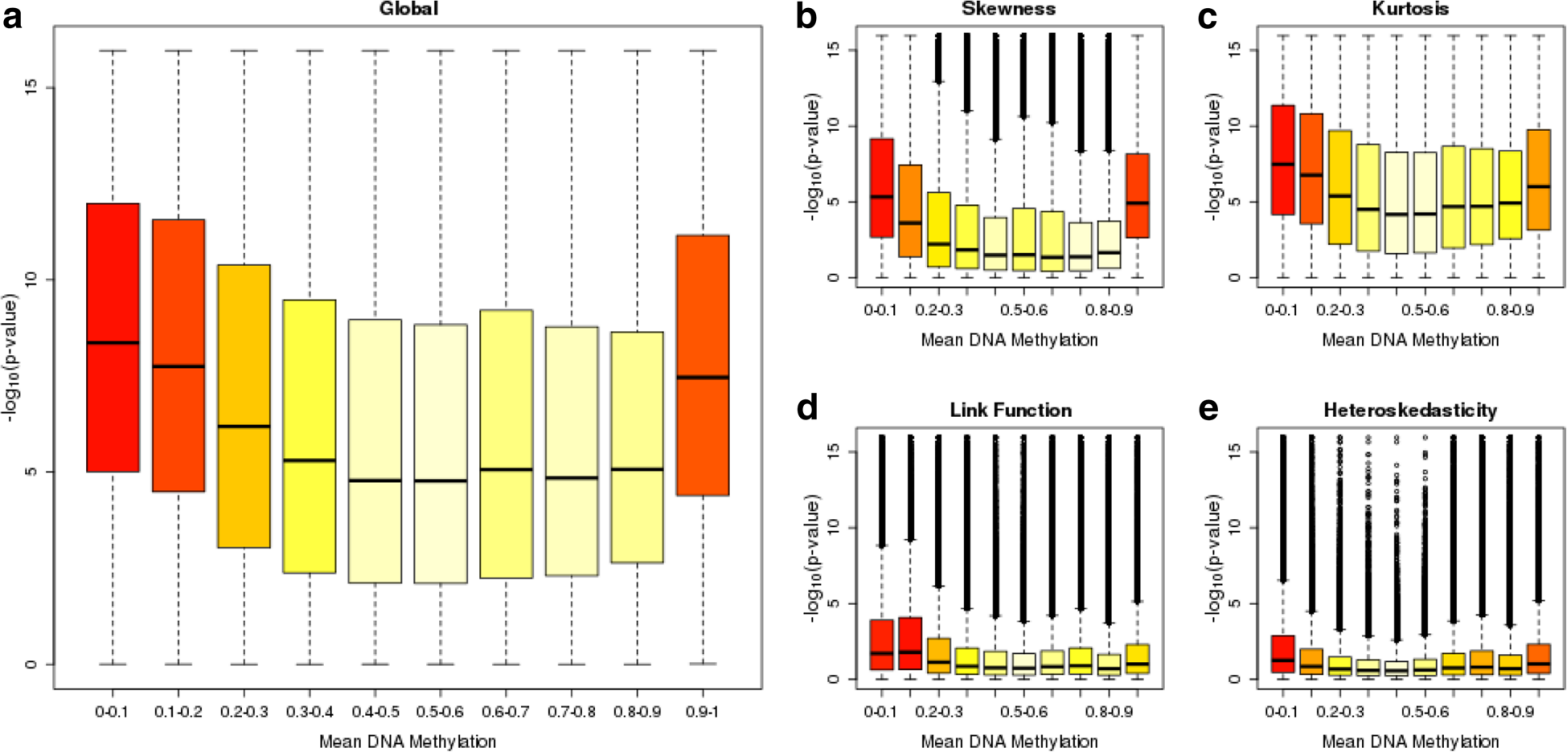
Comparison of tests of linear regression assumptions across the distribution of DNA methylation levels. Boxplots of –log_10_(p-value) for each of the 5 tests (**a**) global (**b**) skewness (**c**) kurtosis (**d**) link function and (**e**) heteroskedasticity for groups of DNA methylation sites binned by their mean DNA methylation level. The boxes are coloured by their mean –log_10_(p-value) from light yellow (low) to red (high)

**Fig. 5:**
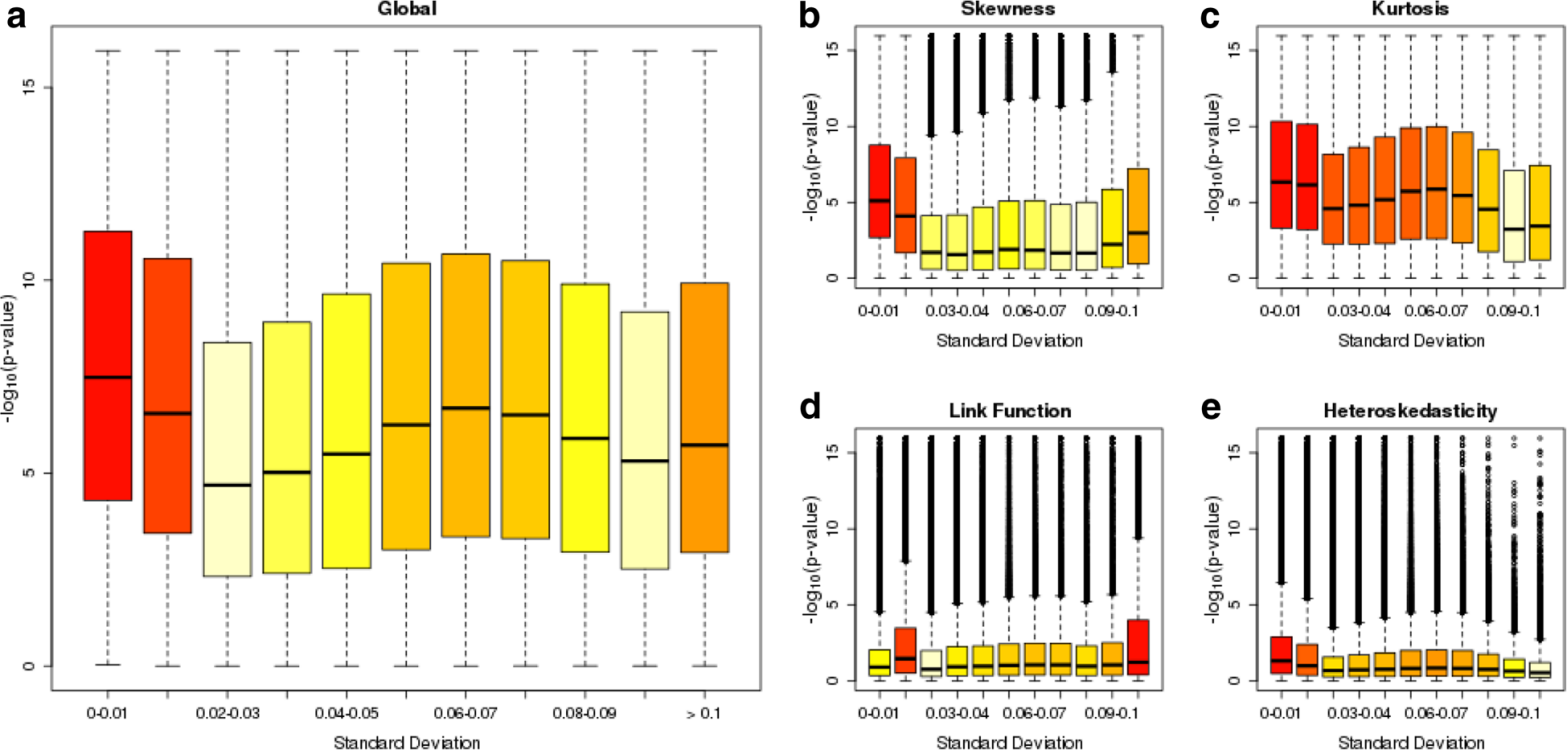
Comparison of tests of linear regression assumptions against DNA methylation variability. Boxplots of –log_10_(p-value) for each of the 5 tests (**a**) global (**b**) skewness (**c**) kurtosis (**d**) link function and (**e**) heteroscedasticity for groups of DNA methylation sites binned by their standard deviation. The boxes are coloured by their mean –log_10_(p-value) from light yellow (low) to red (high)

In order to propose guidelines for future EWAS studies, we were interested in whether DNAm sites that performed poorly in the *gvlma* tests could be characterized by common features such as DNAm level or variability. First, we considered the level of DNAm at each site, hypothesising that sites which are located at the extremes of the distribution would be more likely to violate the assumptions of the tests. We observed that the sites with the most significant *p*-values in the *gvlma* tests (i.e. those with the largest –log10 p-values) are generally either hypo- or hypermethylated (Additional file 1: Figure S4). Furthermore, by grouping sites based on their mean DNAm level we can pinpoint where in the distribution of DNAm values the assumptions are typically not satisfied. We observe a U-shaped relationship whereby sites with DNAm levels at the extremes (i.e. approaching 0 or 1), are more likely to violate the assumptions compared to sites with intermediate levels of DNAm (Fig. 4; Additional file 2: Table S2). This pattern generally holds for all four tests, but is most apparent for tests of skewness and kurtosis. Of interest, the relationship is not symmetrical, with the first two bins on the left of the distribution (containing sites with means of between 0 and 0.2) but only one bin on the far right of the distribution (containing sites with means of between 0.9 and 1.0) showing elevated mean –log10 *p*-value compared to the middle seven bins. Second, we considered site variability, hypothesising that sites with low levels of variation would be more likely to violate the test assumptions. Using the standard deviation to index variability, we observed that sites with lower standard deviations had larger -log10 *p*-values when testing the assumptions of linear regression (Additional file 1: Figure S5). This was most evident for the tests of skewness, kurtosis and heteroskedasticity, in particular for sites with a standard deviation < 0.02 (Fig. 5; Additional file 2: Table S3). A more complex pattern was seen for the link function test, where the most variable probes and the second group of least variable probes had the highest –log10 p-values. Using an alternative non-parametric method to characterize sites as ‘variable’ (range of middle 80% of values > 5%) or ‘non-variable’, we observed a similar pattern of results (Additional file 1: Figure S6; Additional file 2: Table S4) where non-variable sites were more likely to reject the assumptions of linear regression compared to variable sites. Taken together, these findings suggest that sites with extreme DNAm levels or low variation are most likely not to satisfy the assumptions of linear regression. These characteristics are not unrelated because sites with low levels of variation are typically located at the boundaries of the distribution of DNAm (Additional file 1: Figure S7).

Recently, M-values have been proposed as an alternative to beta-values in EWAS analyses of traits and exposures due to their more desirable statistical properties [18]. Although a direct comparison of beta-values and M-values is beyond the scope of this manuscript, we repeated our analyses on M-values to further interpret the results presented above. Using our experiment-wide significance threshold, 70.1% of DNAm sites demonstrated significant bias of at least one assumption when using M-values; that is just 1.09% less than the original analysis based on beta-values (Additional file 2: Table S5). Furthermore, 85.9% of DNAm sites that are considered statistically inappropriate based on beta-values were also classed as statistically inappropriate when analysed as M-values. As with the beta-value analysis, the primary assumption violated by M-values related to the shape of the distribution of residuals. In fact, a comparable number of sites demonstrated excess kurtosis regardless of whether beta-values (67.6%) or M-values (66.7%) were used. Furthermore, albeit more subtly, DNAm sites with methylation levels at the extreme ends of the distribution were more likely to fail the statistical tests (Additional file 1: Figure S8), consistent with the results of the analysis using beta-values.

### Evaluating the impact on DNAm studies of sites that do not meet the assumptions of linear regression

**Fig. 6:**
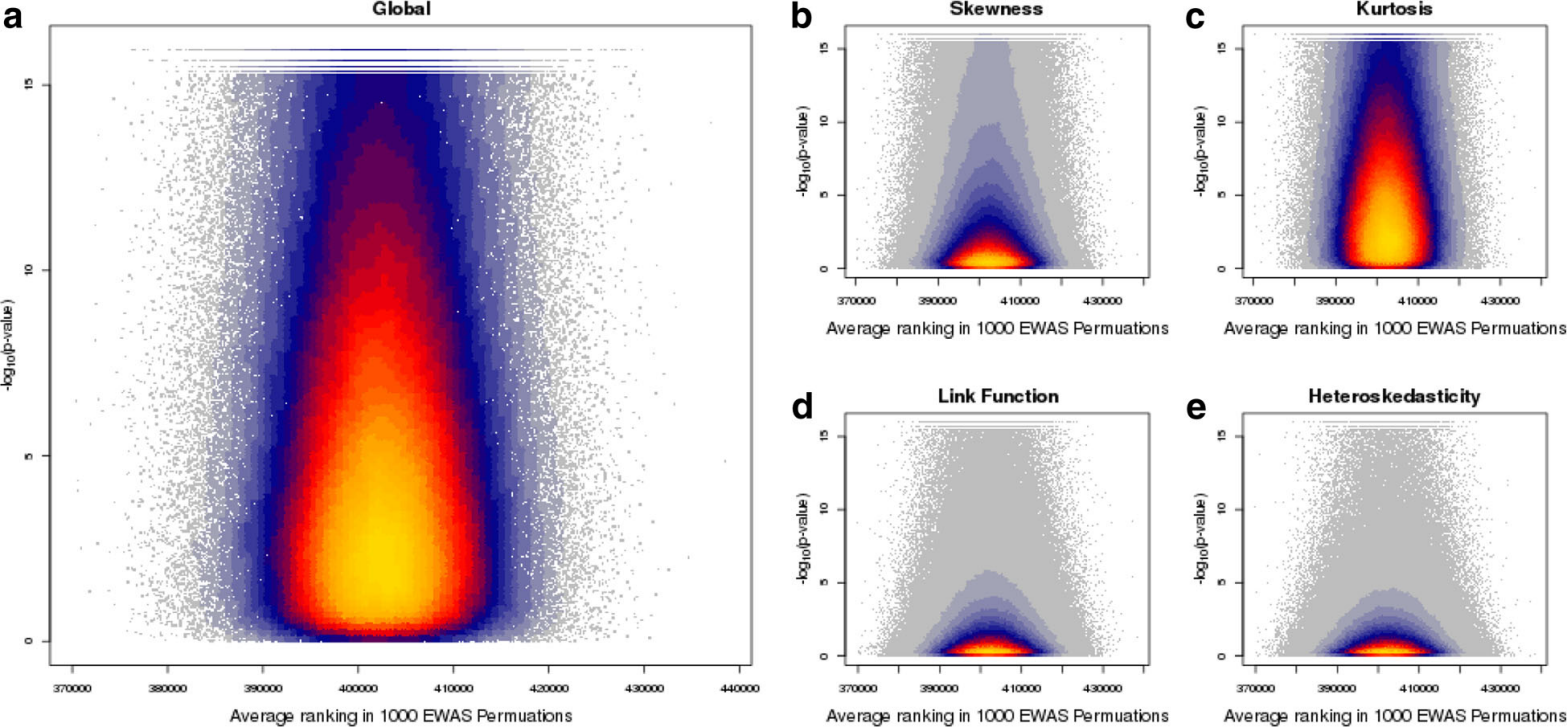
Comparison of tests of linear regression assumptions with bias in DNA methylation association studies. Scatterplots of –log_10_(*p*-value) (y-axis) from the (**a**) global (**b**) skewness (**c**) kurtosis (**d**) link function and (**e**) heteroskedasticity tests performed in the R *gvlma* package against average (mean) ranking from 1000 simulated null association studies (x-axis) for all DNA methylation sites. Each point represents a single site, and the color represents the density of points plotted at that position (low density in grey to high density in yellow)

The primary concern about using an invalid analytical model is the risk of either reporting false positive or false negative findings in tests of association. As linear regression is considered robust to violations of the assumptions, we next explored whether sites that violated an assumption were more likely to be significant in a DNAm analysis using a linear regression model. Using our simulated null association studies, DNAm sites were ranked by their association *p*-value to calculate the mean rank across the simulations. In a scenario where all sites are equally likely to be associated and there is no bias in the analysis, the distribution of these mean ranks should be symmetrical and unimodal with a mean of 402,413.5. Any skew in the distribution, or the presence of outliers and/or multiple peaks, would indicate an underlying bias in which DNAm sites are often identified as significant or not. We found that the distribution of the mean rank was normally distributed with a mean of 402,446 (Additional file 1: Figure S9), similar to the expected value. We observed no association between *p*-values from the *gvlma* tests and a DNAm site’s mean rank indicating that even highly significant rejections of the assumptions of linear regression do not bias EWAS results in terms of either false positives or false negatives (Fig. 6; Additional file 1: Figure S10; Additional file 2: Table S6).

### Estimating the power of an EPIC array DNAm study

**Fig. 7:**
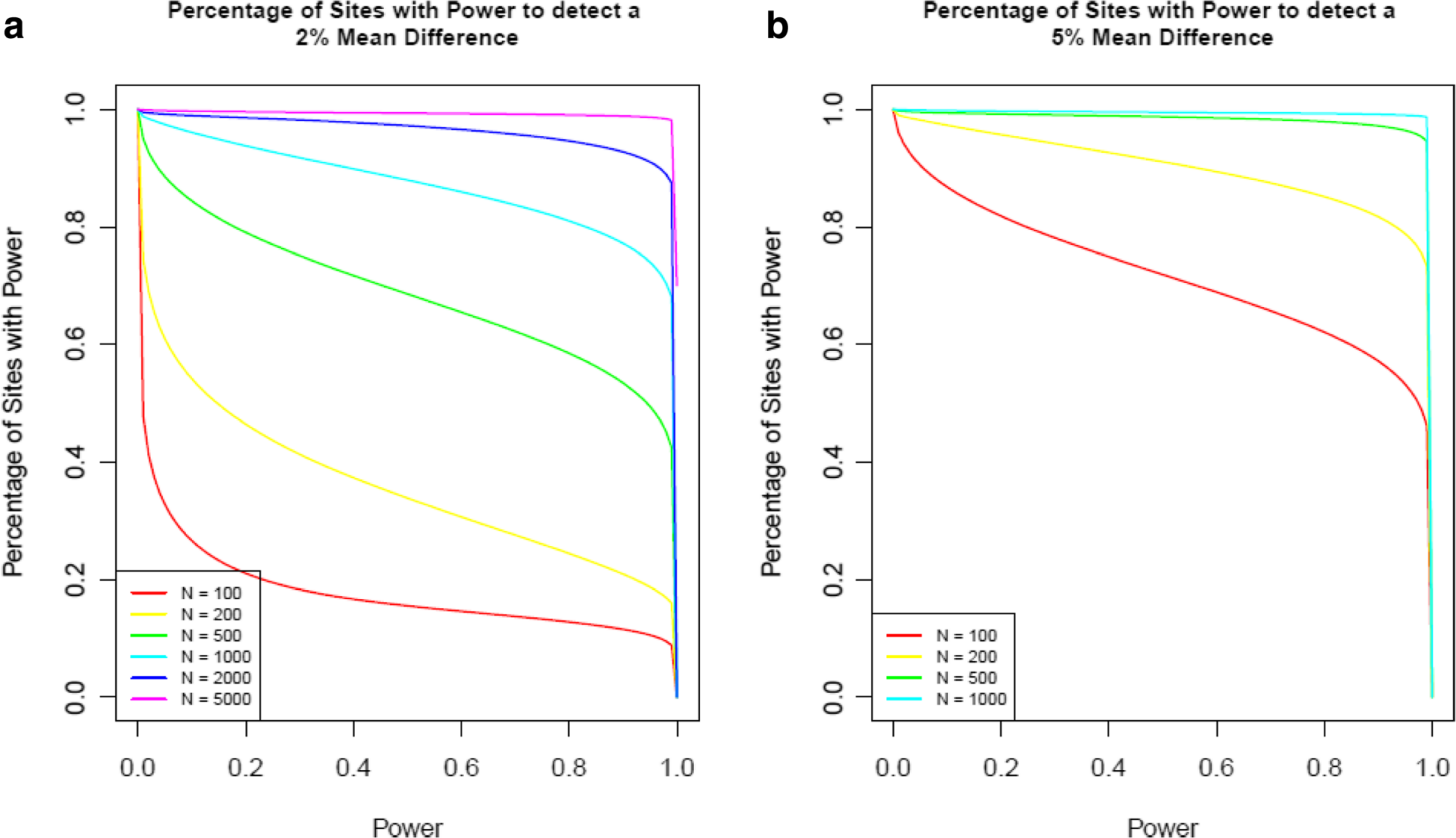
Power curves of typical DNA methylation studies. Line graphs depicting the proportion of sites on the EPIC array (y-axis) with sufficient power (x-axis) to detect a mean difference in DNA methylation between two groups of (**a**) 2% and (**b**) 5%. The different coloured lines represent different sample sizes where the value of N the total sample size set to be a 50:50 split between groups

The power of a test is defined as the probability that it correctly rejects the null hypothesis when the alternative hypothesis is true. As with other types of genomic analyses, large sample sizes are required for EWAS in order to obtain the statistical power required to identify a significant non-zero effect with a p-value that survives the adjustment for multiple testing. Having derived an appropriate multiple testing corrected significance threshold for the EPIC array, we investigated the typical sample sizes required for a DNAm study using this platform. In order to estimate power we need to know the sample size, multiple testing threshold, expected effect size and variance. While the first three of these parameters will remain constant for a particular study, the variance of DNAm will vary across sites. This means that a single power calculation, perhaps based on an average probe, provides limited information about the overall power of a DNAm study. We therefore performed a power calculation for each individual site on the EPIC array and then established the proportion of sites that surpass a specific power threshold. The estimated power for a single association test across a range of standard deviations and sample sizes for a binary phenotype (as would be tested in a disease case-control study) are shown in Table 2. For example, to detect a mean difference of 2% with 500 cases and 500 controls (total *N* = 1000), we have 100% power at sites with a standard deviation <= 0.03. Performing separate power calculations tailored by the variance of each site, we plotted power curves for a range of typical DNAm studies (Fig. 7). This analysis demonstrates that when *N* = 200 (100 cases and 100 controls), 85% of sites have > 80% power to detect an effect of 5% (yellow line in Fig. 7b), and when N = 1000 (500 cases and 500 controls), 81% of probes have > 80% power to detect an effect of 2% (light blue line in Fig. 7a). While these examples provide a general overview of power for EPIC array studies, the results are also available for browsing in an interactive web application (https://epigenetics.essex.ac.uk/shiny/EPICDNAmPowerCalcs/) where the parameters can be adjusted in order to generate bespoke power calculations allowing researchers to assess the power of their individual study.

**Table 2:** Summary of statistical power to significantly detect differential methylation between cases and controls

Sample Size	Standard Deviation					
	0.01	0.03	0.05	0.07	0.09	0.15
Mean Difference = 2%						
100	100%	1.26%	0.03%	0.00%	0.00%	0.00%
200	100%	21.4%	0.45%	0.04%	0.01%	0.00%
500	100%	97.8%	17.6%	1.43%	0.19%	0.01%
1000	100%	100%	82.7%	19.7%	3.22%	0.06%
Mean Difference = 5%						
100	100%	99.1%	24.4%	2.18%	0.29%	0.01%
200	100%	100%	93.0%	32.0%	6.07%	0.11%
500	100%	100%	100%	99.4%	78.4%	4.81%
1000	100%	100%	100%	100%	100%	45.8%

Presented are example power calculations for a range of scenarios, varying effect size, sample size and variance for a binary phenotype. Power calculations are for a two-sided, two-sample t-test with a significance threshold of P < 9.42 × 10^− 8^. The sample size is the total number of samples with a 50:50 split between groups

## Discussion

This study used a large DNAm dataset generated using the Illumina EPIC array to assess the statistical properties that influence the analytical design for hypothesis testing in epigenome-wide association studies. We estimated that there are 530,639 independent tests in a whole blood EPIC array DNAm study, which equates to a corrected significance threshold of 9.42 × 10^− 8^. For ease, we propose 9 × 10^− 8^ would be an appropriate EPIC array experiment-wide significance threshold that should be adopted by the field to minimize the reporting of false positives. Although this EPIC array experiment-wide threshold is not substantially different to a Bonferroni correction for the actual number of tests, our estimate is comparable to that proposed using a similar methodology to data from the older 450 K array [32], which includes approximately half the number of sites (*P* = 2.4 × 10^− 7^) that were converted to M-values. Our results indicate that the correlation in DNAm across sites included on the Illumina EPIC array is relatively small and does not encompass large genomic regions; Saffari and colleagues also observed that strong correlations between neighboring sites were typically only observed within 1 kilobase [32], consistent with the minimal reduction from number of actual tests to number of independent tests we report. This challenges the argument that a Bonferroni correction is too conservative and therefore a more relaxed multiple testing threshold can be applied. Existing and future studies which report results at a more lenient threshold, particularly those with small sample sizes and lower statistical power should be interpreted with caution.

We attempted to extrapolate from the experiment-wide threshold for the EPIC array to estimate an appropriate threshold for all potential tests across the genome, including those not currently profiled by the EPIC array, by using simulations to profile how the number of independent tests changes as the coverage of the microarray increases. At sufficient density, the number of independent tests should plateau; however this behaviour was not really evident across the range of densities we were able to simulate, suggesting that the EPIC array does not interrogate a large part of the variation in DNAm across the genome. Therefore, our estimate of the number of independent tests in the genome is likely to be imprecise. Moreover, given the wide confidence interval around the estimated genome-wide multiple testing burden, we recommend this result is taken with some caution. Future large population based studies that include more DNAm sites across the genome would be required to address this question. We propose that our experiment-wide significance threshold should be adopted for all future EPIC array EWAS. The use of a standardized significance threshold would benefit the field by providing a common standard for reporting associations and facilitate the comparison of results across different studies. While the threshold has been determined to minimize the reporting of false positives, it does not prevent them entirely; prudent study design and effective control of confounders are still required for high quality EWAS studies. Furthermore, replication of associations across independent datasets is still required to validate robust associations.

We also tested the assumptions of linear regression, the most commonly used tool for identifying associations between differential DNAm and a trait, when measuring DNAm using beta-values (i.e. as a proportion) and conclude that the majority of sites do not satisfy the assumption of normally distributed errors. This was particularly the case for DNAm sites that have low levels of variation or are located at the extreme ends of the distribution. While we use our experiment-wide *p*-value threshold to quantify the number of probes not satisfying these assumptions in order to gauge the pattern of results, we caution against using this threshold to classify sites as passing or failing these assumptions. As the statistical evidence required to reject the null hypothesis in these tests is unlikely to equate to the degree of violation of the assumption needed to influence the results of the regression analysis, it may not follow that sites that fail these tests will lead to incorrect conclusions if a linear regression model is used. As these assumptions were tested on an EWAS of chronological age, it is possible that our results are specific to this particular analysis. Furthermore, we used a European adult whole blood cohort as a basis for our assessment, which may mean that the results are not applicable to studies of other tissues, cell-types, ages or ethnicities. It is also likely that these violations of these assumptions will be more important for studies based on smaller sample sizes. For these reasons, rather than report a list of DNAm sites that do not satisfy the assumptions, we focused on characterising these sites in order to provide general guidelines. Although the specific sites that not do vary within a sample may differ between studies, we predict that it is always the non-variable sites that fail the tests of the assumptions. Some studies remove non-variable sites prior to hypothesis testing [38–40] and our results support such a filtering step. However, as we found no evidence that the lack of normal residuals, an incorrectly specified link function, or heteroskedasticity leads to either false positive or false negative associations, our data also suggests that this is not strictly necessary. A number of studies have used transformations of beta-values, for example using log ratios of methylation percentage referred to as M-values in order to obtain a normal distribution [3, 10, 18] or regression based on an alternative distribution (e.g. beta regression [41]); our results show that the use of linear regression with beta values in DNAm studies, even if the data do not satisfy the standard assumptions of this test, does not appear to lead to biased results. Despite considering the four key assumptions of linear regression, we did not specifically investigate the effect of outlier DNAm values, which may arise due to either technical or biological artefacts (e.g. rare SNP effects). The presence of outliers can introduce false positive associations as linear regression estimates are derived by minimising the sum of the residuals, therefore extreme values, which would lead to large residuals, can lead to larger, and therefore significant, estimated slope coefficients.

Finally, we performed power calculations to ascertain the sample size required for EPIC array studies using our proposed experiment-wide significance threshold. Most complex phenotypes are expected to be associated with small effects (typically < 5% difference between cases and controls), and our calculations indicate that with a sample size of 500 cases and 500 controls, 81% of sites have > 80% power to detect an effect of 2%. This estimate should be reassuring to the epigenetic community, as there are an increasing number of studies approaching or surpassing this sample size [9, 42–45]. Our approach advances previous efforts [46] by taking into account the individual properties of each DNAm site and uses an empirically derived significance threshold to provide an overview of power across the EPIC array. Finally, we have developed an online tool (https://epigenetics.essex.ac.uk/shiny/EPICDNAmPowerCalcs/) where users can perform their own bespoke calculations to quantify the power of their specific study for individual DNAm sites; we are currently extending this power calculation application for use with quantitative trait variables, and will implement an updated version in the near future.

## Conclusions

We show that linear regression is a valid statistical methodology for DNAm studies, despite the fact that the data do not always satisfy the assumptions of the test. Additionally, we propose that a significance threshold of *P* < 9 × 10^− 8^ should be adopted to adequately control the false positive rate for EPIC array based analyses and should be accepted as a standard for reporting results. These findings have implications for epidemiological-based DNAm studies and provide a framework for the interpretation of findings from current and future studies.

## Methods

All analyses were performed using the statistical language R [47].

### Genomic-wide profiling of DNAm in understanding society

The DNAm dataset generated as part of the Understand Society study has been analysed in two previously published studies [34, 48] and a detailed description of the sample, DNAm data generation and data preprocessing can be found in the original publication [34]. Briefly, Understanding Society (https://www.understandingsociety.ac.uk) is a longitudinal panel survey of 40,000 UK households which has collected sociodemographic information, biomedical measures and blood samples from participants. DNA was extracted from whole blood samples to facilitate genomic profiling including DNAm.

### DNAm data preprocessing

DNAm was profiled for a subset of 1193 individuals from the Understanding Society study using the Illumina Infinium HumanMethylationEPIC BeadChip. Raw signal intensity data were processed from idat files through a standard pipeline using the bigmelon [48] and wateRmelon [49] packages in R. A number of quality control steps were performed to these data prior to normalization. First, outlier samples were identified using principal component analysis and mahalanobis distance equivalents, second, successful bisulphite conversion was confirmed using control probes, third the ages of the samples were estimated using the Horvath Epigenetic Clock algorithm [50] and compared to reported age at sampling, and fourth visualisation of principal components. These data were then normalized using the *dasen* method [49], which performs background adjustment and between-sample quantile normalization of methylated (M) and unmethylated (U) intensities separately for Type I and Type II probes. A second round of sample filtering was then performed excluding samples that were either dramatically altered as a result of normalisation or samples that had > 1% of sites with detection *p*-value > 0.05. DNAm sites were filtered to exclude those with a bead count < 3 or > 1% of samples with detection p-value > 0.05. The raw DNAm data of the final sample set was then re-normalized with the *dasen* method. Prior to data analysis, SNP probes, probes with non-specific binding, probes affected by common SNPs [51], and 65 probes annotated to the Y chromosome were additionally removed. The final dataset contained 1175 individuals and 804,826 DNAm sites (787,400 annotated to autosomes, and 17,426 annotated to the X chromosome).

### Estimating a significance threshold for DNAm studies using the EPIC array

To estimate an experiment-wide significance threshold for the EPIC array, we applied the permutation procedure previously described by Dudbridge and Gusnanto [35]. For each permutation, 50% of our 1175 samples (*n* = 557) were randomly assigned as “cases” and 50% (*n* = 558) as “controls” to simulate a null EWAS (i.e. no differences between cases and controls). Each of the 804,826 sites was then tested for association with this simulated phenotype using a linear regression model including sex, age, and six estimated cellular composition variables (B cells, CD8 T cells, CD4 T cells, monocytes, granulocytes, natural killer T cells) [52, 53] as covariates. We repeated this procedure 1000 times recording the smallest *p*-value (i.e. the most significant) from each permutation. The EPIC array significance threshold was estimated by taking the 5th percentile point of these 1000 minimum *p*-values representing the 5% family-wise error rate (FWER).

### Estimating a genome-wide significance threshold for DNAm studies

In order to extrapolate from our experiment-wide significance thresholds to one appropriate for genome wide DNAm association studies, we implemented the subsampling procedure also implemented by Dudbridge and Gusnanto [35]. Briefly, to simulate experiments with a reduced number of sites that capture a smaller proportion of genome-wide variation, sites were randomly subsampled at a range of densities (*x*_*i*_ = 5, 15%, …, 95%; *i* = 1, 2, …, 10). From each permutation, the smallest p-value across the subset of sites was extracted and the 5th percentile point across all 1000 minimum p-values was recorded. This subsampling was repeated 100 times and the mean, 2.5 and 97.5 percentile points were calculated to set the significance threshold (P_T*i*_) and confidence intervals for density *i*. At low densities, where the coverage is sparse, it is assumed that all included DNAm sites will be independent and a Bonferroni correction for multiple testing is appropriate. As coverage increases, correlations between neighboring sites mean that the number of additional independent tests decreases. In other words, continually increasing the number of sites studied has diminishing returns in terms of the increase in additional variation captured. Therefore, as the number of sites profiled in an experiment increases, the effective number of independent tests converges to an asymptote. To estimate the value of this asymptote, we fitted a Monod function across the site densities and their estimated number of independent tests. For each of the site densities (*x*_*i*_), the effective number of independent tests (*m*_*i*_), was calculated by using the inverse of the Bonferroni correction for multiple testing (*m*_*i*_ = 0.05/P_T*i*_). A Monod function, originally a mathematical model for bacterial population growth with limited resources, takes the form:

$$ m=f\left(x,u,k\right)=\frac{ux}{k+x} $$

where *u* is the limit and *k* is the half-saturation parameter, their values given by:

$$ f(k)=\frac{u}{2} $$

$$ f\left(\infty \right)=u $$

This function was fitted using a least squares approach in R to find the value of *u*, which represents the number of independent tests in the entire DNA methylome. To calculate the methylome-wide significance threshold we applied the Bonferroni correction using this estimate (P_genome_ = 0.05/*u*).

### Testing the assumptions of linear regression models used in DNAm studies

To assess the validity of linear regression models in studies of DNAm, an EWAS of age was performed including sex, processing chip and six estimated cellular composition variables (B cells, CD8 T cells, CD4 T cells, monocytes, granulocytes, natural killer T cells) [52, 53] as covariates. For each of the 804,826 models (one per DNAm site) we tested for violations of the assumptions of linear regression using the *gvlma* (Global Validation of Linear Model Assumptions) R package [37]. This package performs four tests to test the performance of the model fit with regards to the four assumptions of a linear regression: linearity, homoskedasticity, uncorrelatedness and normality of the residuals (Additional file 1: Figure S2). The *gvlma* package provides a numerical measure of violation through significance testing for skewness, kurtosis, link function, and heteroskedasticity. Briefly, the package calculates a directional test statistic for each assumption using the standardized residuals from the fitted linear model. These test statistics are each compared to a 1 degree-of-freedom chi-square distribution to calculate a *p*-value for hypothesis testing. In addition to obtaining a p-value for each of these four tests, the software also generates a “global” p-value, which is an omnibus test of the four others. The global test statistic is the sum of the four components (one for each assumption) and compared to a 4 degree-of-freedom chi-square distribution. The formula for each component and further details can be found in the original manuscript proposing the method [37]. The null hypothesis for the global test is that all four assumptions hold, and the alternative hypothesis is that at least one does not (i.e. a significant p-value indicates that a linear model is not appropriate). In order to assess how DNAm sites on the EPIC array performed across these five tests we plotted Quantile-Quantile (QQ) plots of the observed vs expected *p*-values. To characterize sites which perform poorly in these tests we visualized correlations between the p-values from the five *gvlma* tests and both the mean level of DNAm and two measures of variance (standard deviation and range of the middle 80% of values). For the purpose of assessing which assumptions are most commonly violated, and which are most commonly violated simultaneously, we applied the experiment wide p-value threshold derived in the previous sections (*P* < 9.42 × 10^− 8^), to identify sites that reject the assumptions of linear regression. Finally to investigate the impact of violating the assumptions of linear regression, we calculated the mean rank across the 1000 null EWAS permutations as an indicator of how likely a site was to be associated by chance and any bias in association analyses. These mean ranks were then compared with the p-values of the *gvlma* tests.

### Estimating statistical power for EPIC array studies

Power calculations were performed for each of the 804,826 sites in the dataset using the function *pwr.t.test* from the R package pwr [54]. We consider the scenario with a binary outcome (i.e. case control study), using a two-sample t-test to compare the means of the two groups where the null hypothesis of each test is that the means of the two groups are equal. To calculate power, the parameters sample size, effect size and significance level were provided. The significance level was set as our previously calculated experiment-wide threshold of 9.42 × 10^− 8^. The effect size was provided as Cohen’s d, which is the expected difference between the two group means divided by their pooled standard deviation [55]. In order to get a power estimate for the overall study, calculations were performed for every site individually using that site’s variance estimated from the Understanding Society dataset, for two different mean differences (2 and 5%). Power calculations were also performed for a range of total sample sizes (*n* = 100, 200, 500, 1000, 2000 and 5000) consisting of equal numbers of cases and controls. For each combination of parameters (sample size and mean difference), we calculated the percentage of sites that had sufficient statistical power across the full range of possible values (0–100%). While we only present results for a subset of the possible scenarios as a guide to the power of a typical EWAS study, we have also developed an R shiny app [7] to allow users to perform bespoke power calculations (https://epigenetics.essex.ac.uk/shiny/EPICDNAmPowerCalcs/). In this app, the user can specify sample size and mean difference to generate a summary results table and downloadable figure. As performing > 800,000 power calculations is time consuming, the app uses a binning method, grouping sites with similar variances and plotting a smoothed curve, to speed up the calculation. For more accurate results the user can increase the number of bins, or chose to calculate the power for all sites individually. There is also an option to search for a specific DNAm site of interest and calculate its power under user defined parameters.

## Additional files

Additional file 1: **Figure S1.** Distribution of minimum *p*-values from 1000 null EWAS simulations. The red line at 9.42 × 10^–8^ is the 5th percentile point and represents the 5% FWER. **Figure S2.** The four assumptions of linear regression (described in the left boxes), were tested using R package *gvlma* with four statistical tests (described in the right boxes) with arrows matching the assumptions to the relevant test. *Gvlma* calculates a *p*-value for each test, where the null hypothesis is that the assumption(s) hold true, and the alternative hypothesis is that they do not (i.e. a significant p-value indicates that the assumption(s) are violated). **Figure S3.** Quantile-Quantile plots of the 5 tests of the assumptions of linear regression. Plotted are the observed (x-axis) against expected (y-axis) –log_10_(p-value) from the **(a)** global **(b)** skewness **(c)** kurtosis **(d)** link function and **(e)** heteroskedasticity tests performed in the R *gvlma* package for all DNA methylation sites. Under the null distribution, of no significant associations, all points would be expected to lie on the red line at y = x. The observed data show a dramatic inflation of *p*-values smaller than expected by chance in all 5 plots indicating that many DNA methylation sites fail these statistical tests for the assumptions of linear regression. **Figure S4.** Scatterplots of –log_10_(*p*-value) against mean DNA methylation level from the **(a)** global **(b)** skewness **(c)** kurtosis **(d)** link function and **(e)** heteroskedasticity tests performed in the R *gvlma* package for all DNA methylation sites. Each point represents a single site, and the color of the point represents the density of points plotted (low density in grey to high density in yellow). **Figure S5.** Scatterplots of DNA methylation standard deviation against –log_10_(*p*-values) from the **(a)** global **(b)** skewness **(c)** kurtosis **(d)** link function and **(e)** heteroskedasticity tests performed in the R *gvlma* package. Each point represents a single DNA methylation site, and the color of the points represents the density of points plotted (low density in grey to high density in yellow). **Figure S6.** Boxplots of –log_10_(*p*-value) for each of the 5 tests: **(a)** global **(b)** skewness **(c)** kurtosis **(d)** link function and **(e)** heteroscedasticity separated by site variability status. DNA methylation sites were defined as variable if the range of their middle 80% of values, calculated as the 90th percentile (P_90_) minus the 10th percentile (P_10_) was greater than 5%. Each boxplot is colored by their mean –log_10_(p-value), from light yellow (lowest –log_10_(p-value)) to red (highest –log_10_(p-value). Sites with a p-value of 0 (i.e. *p* < 2.22 × 10^–16^) were removed from these plots. **Figure S7.** Scatterplot of variability (standard deviation; y-axis) against mean methylation level (x-axis), for all DNA methylation sites tested. The color of the points represents the density of points plotted (low density in grey to high density in yellow). **Figure S8.** Comparison of suitability of linear regression assumptions for M-values across the distribution of DNA methylation levels. Boxplots of –log_10_(p-value) for each of the 5 tests **(a)** global **(b)** skewness **(c)** kurtosis **(d)** link function and **(e)** heteroscedasticity for groups of DNA methylation sites binned by their mean DNA methylation level, measured as a beta-value. The boxes are coloured by their mean –log_10_(p-value) from light yellow (low) to red (high). **Figure S9.** Histogram of DNA methylation sites mean rank from simulated null association studies. The red vertical line indicates the expected value under the scenario of no bias of 402,413.5. **Figure S10.** Boxplots of –log_10_(p-value) for each of the 5 tests: **(a)** global **(b)** skewness **(c)** kurtosis **(d)** link function and **(e)** heteroskedasticity, for groups of DNA methylation sites binned by their mean ranking from our simulations of null association studies. Sites were allocated to nine bins based on their average (mean) rank. Each boxplot is coloured based on their by their mean –log_10_(*p*-value) using a scale from light yellow to red in each subplot. This shows that there is generally no trend between significant *p*-values and mean rank. Sites with a p-value of 0 (i.e. p < 2.22 × 10^–16^) were removed for these plots. (PDF 591 kb)

Additional file 2: **Table S1.** Estimating the multiple testing correction significance threshold for sub-samples of EPIC array DNA methylation sites. **Table S2.** Summary of results from tests of assumptions of linear regression separated by mean DNA methylation level. The number and percentage of DNA methylation sites significant for each test (*P* < 9.42 × 10^–8^) split by mean DNA methylation level. **Table S3.** Summary of results from tests of assumptions of linear regression separated by DNA methylation standard deviation. The number and percentage of DNA methylation sites significant for each test (P < 9.42 × 10^–8^) split by DNA methylation level standard deviation. **Table S4.** Summary of results from tests of assumptions of linear regression separated by DNA methylation variability status. The number and percentage of DNA methylation sites significant for each test (P < 9.42 × 10^–8^). Variable DNA methylation sites are defined as those with the range of their middle 80% of values greater than 5%. **Table S5.** Summary of DNA methylation sites significantly rejecting the assumptions of linear regression comparing beta-values and M-values. For each of the 5 tests performed by the *gvlma* package the number and percentage of DNA methylation sites with significant p-values (P < 9.42 × 10^–8^) are reported for linear regression models based on beta-values and M-values. **Table S6.** Summary of results from tests of assumptions of linear regression separated by mean rank in null association studies. The number and percentage of DNA methylation sites significant for each test (P < 9.42 × 10^–8^) split by their mean rank across 1000 simulated null association studies. (PDF 153 kb)
