# Supplementary material for: Guidance for DNA methylation studies: statistical insights from the Illumina EPIC array
Source: BMC Genomics. 2019 May 14;20:366. doi: 10.1186/s12864-019-5761-7 (PMC6518823; doi:10.1186/s12864-019-5761-7)
Supplement: Supplementary file 1 — Figure S1. Distribution of minimum p-values from 1000 null EWAS simulations. The red line at 9.42 × 10^–8^ is the 5th percentile point and represents the 5% FWER. Figure S2. The four assumptions of linear regression (described in the left boxes), were tested using R package gvlma with four statistical tests (described in the right boxes) with arrows matching the assumptions to the relevant test. Gvlma calculates a p-value for each test, where the null hypothesis is that the assumption(s) hold true, and the alternative hypothesis is that they do not (i.e. a significant p-value indicates that the assumption(s) are violated). Figure S3. Quantile-Quantile plots of the 5 tests of the assumptions of linear regression. Plotted are the observed (x-axis) against expected (y-axis) –log10(p-value) from the (a) global (b) skewness (c) kurtosis (d) link function and (e) heteroskedasticity tests performed in the R gvlma package for all DNA methylation sites. Under the null distribution, of no significant associations, all points would be expected to lie on the red line at y = x. The observed data show a dramatic inflation of p-values smaller than expected by chance in all 5 plots indicating that many DNA methylation sites fail these statistical tests for the assumptions of linear regression. Figure S4. Scatterplots of –log10(p-value) against mean DNA methylation level from the (a) global (b) skewness (c) kurtosis (d) link function and (e) heteroskedasticity tests performed in the R gvlma package for all DNA methylation sites. Each point represents a single site, and the color of the point represents the density of points plotted (low density in grey to high density in yellow). Figure S5. Scatterplots of DNA methylation standard deviation against –log10(p-values) from the (a) global (b) skewness (c) kurtosis (d) link function and (e) heteroskedasticity tests performed in the R gvlma package. Each point represents a single DNA methylation site, and the color of the points represents [file 12864_2019_5761_MOESM1_ESM.pdf]

Additional File 1: Supplementary figures accompanying "Guidance for DNA methylation studies: Statistical insights from the EPIC array"

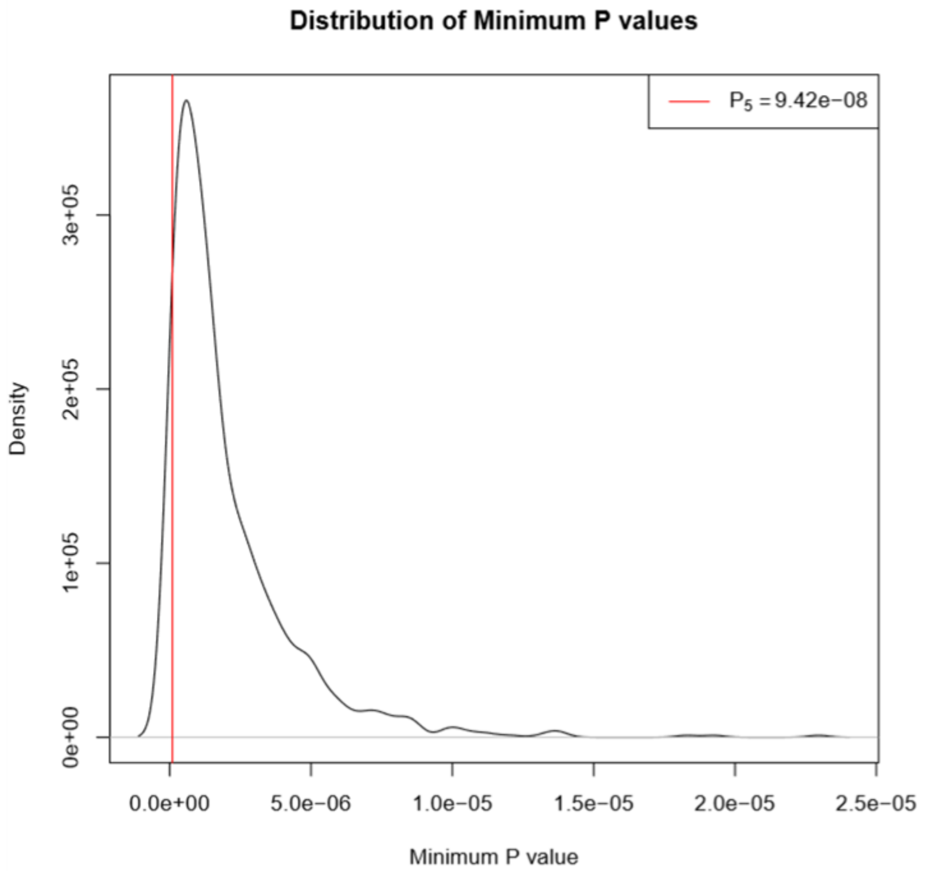

Figure S1: Distribution of minimum p-values from 1000 null EWAS simulations. The red line at 9.42×10^{-8} is the 5th percentile point and represents the 5% FWER.

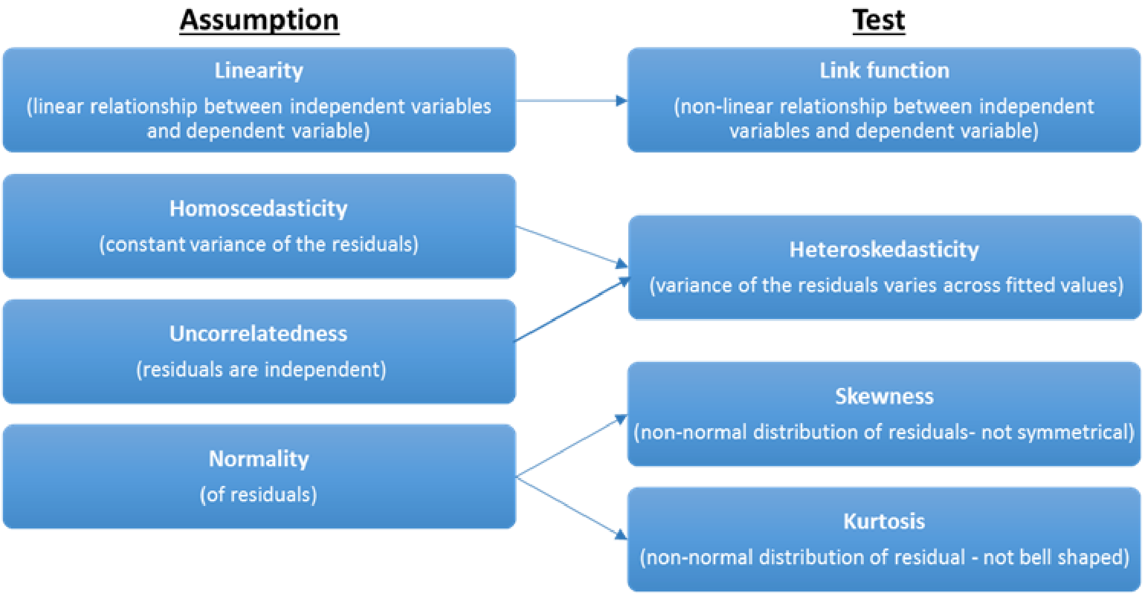

Figure S2: The four assumptions of linear regression (described in the left boxes), were tested using R package *gvlma* with four statistical tests (described in the right boxes) with arrows matching the assumptions to the relevant test. *gvlma* calculates a p-value for each test, where the null hypothesis is that the assumption(s) hold true, and the alternative hypothesis is that they do not (i.e. a significant p-value indicates that the assumption(s) are violated).

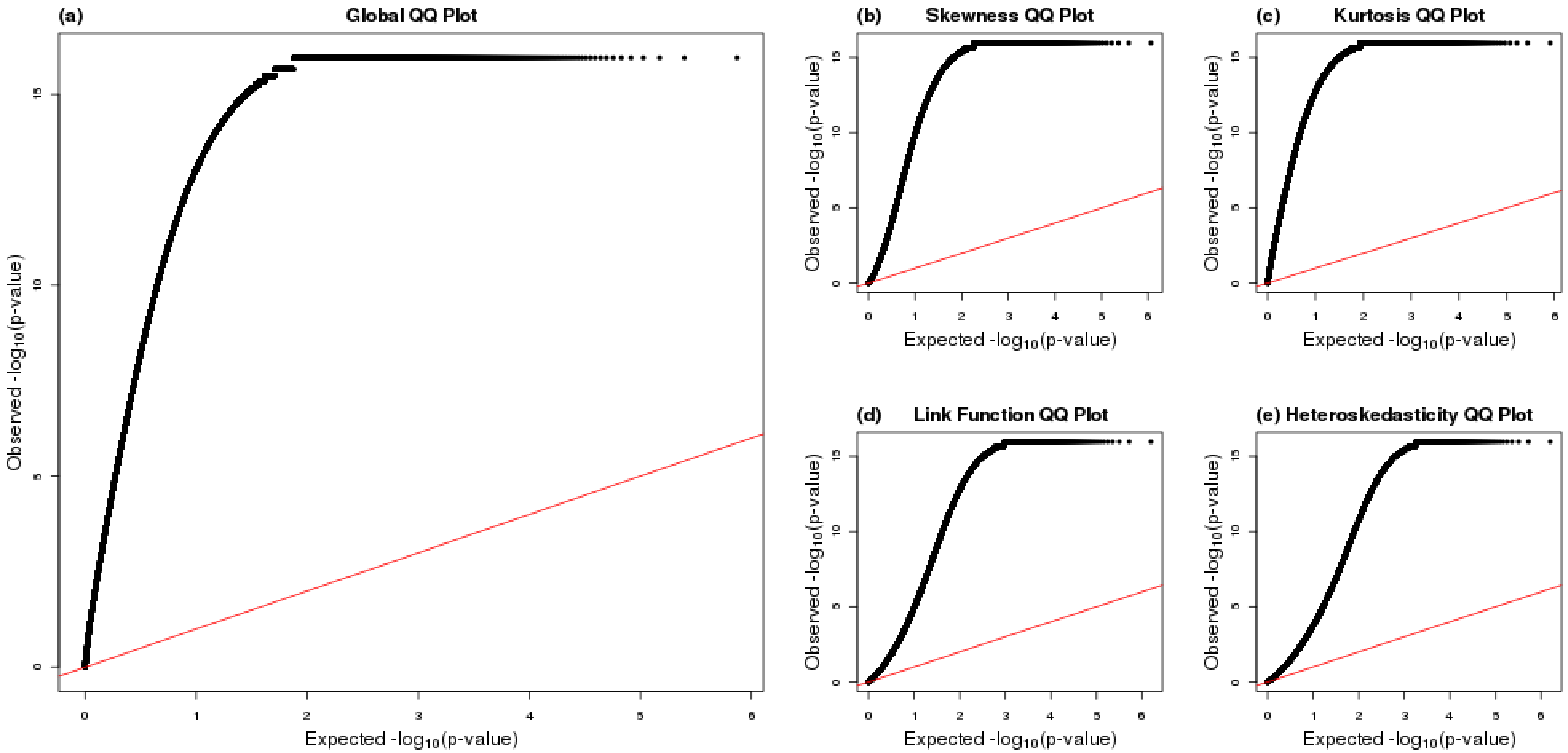

Figure S3: Quantile-Quantile plots of the 5 tests of the assumptions of linear regression. Plotted are the observed (x-axis) against expected (y-axis) $-\log_{10}$(p-value) from the **(a)** global **(b)** skewness **(c)** kurtosis **(d)** link function and **(e)** heteroskedasticity tests performed in the R *gvlma* package for all DNA methylation sites. Under the null distribution, of no significant associations, all points would be expected to lie on the red line at y=x. The observed data show a dramatic inflation of p-values smaller than expected by chance in all 5 plots indicating that many DNA methylation sites fail these statistical tests for the assumptions of linear regression.

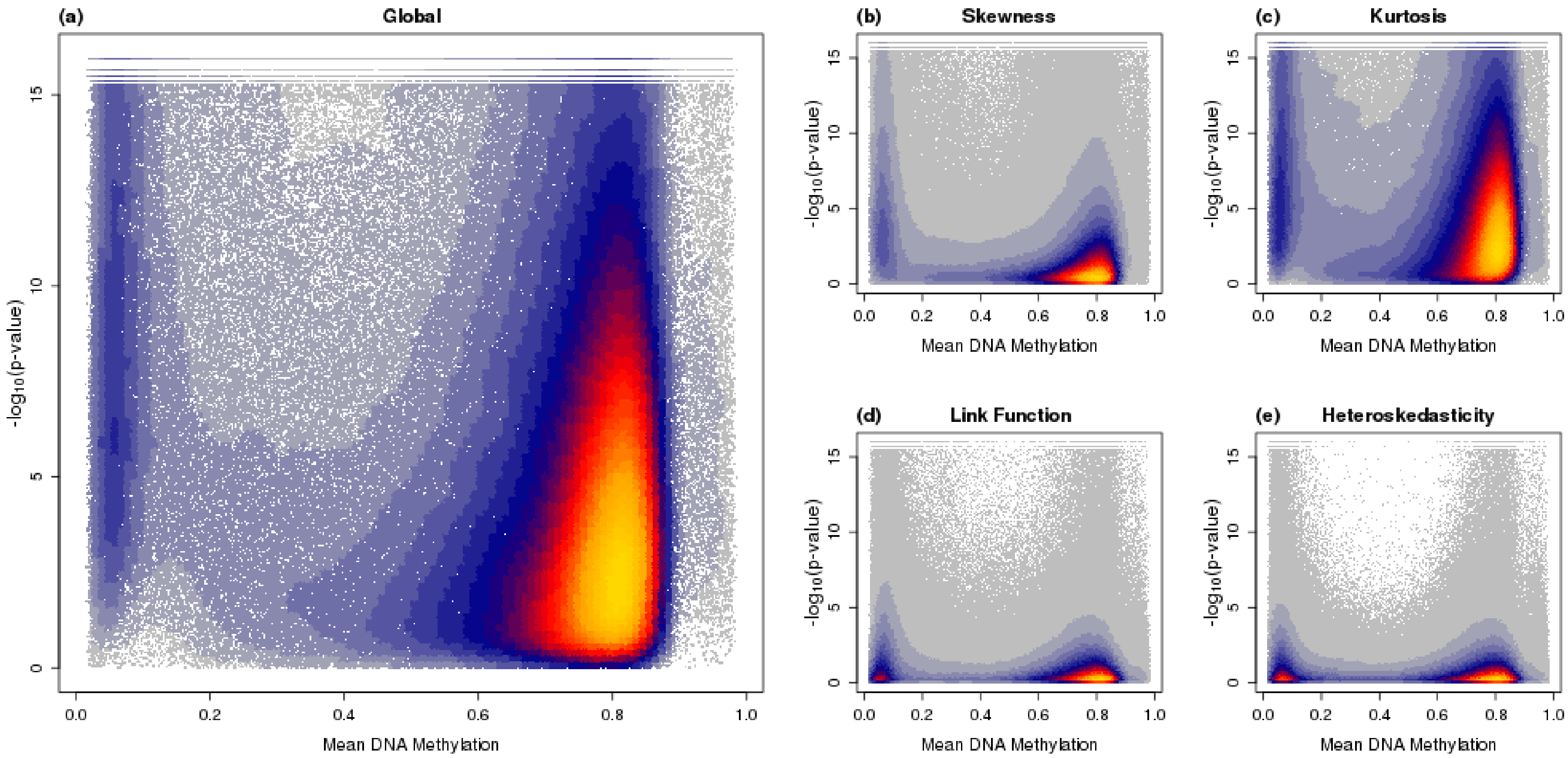

Figure S4: Scatterplots of $-\log_{10}$(p-value) against mean DNA methylation level from the **(a)** global **(b)** skewness **(c)** kurtosis **(d)** link function and **(e)** heteroskedasticity tests performed in the R *gvlma* package for all DNA methylation sites. Each point represents a single site, and the color of the point represents the density of points plotted (low density in grey to high density in yellow).

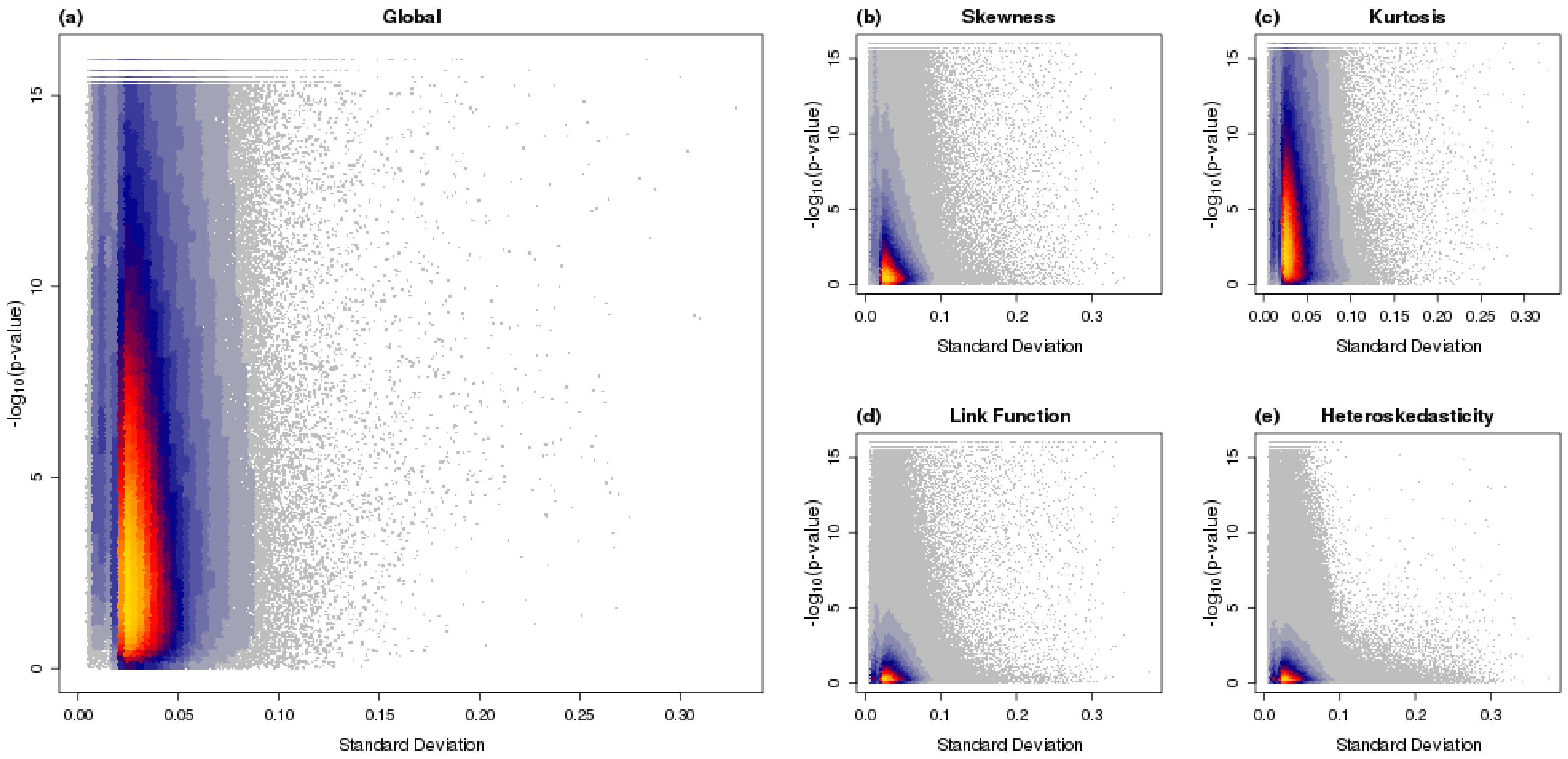

Figure S5: Scatterplots of DNA methylation standard deviation against $-\log_{10}$(p-values) from the **(a)** global **(b)** skewness **(c)** kurtosis **(d)** link function and **(e)** heteroskedasticity tests performed in the R *gvlma* package. Each point represents a single DNA methylation site, and the color of the points represents the density of points plotted (low density in grey to high density in yellow).

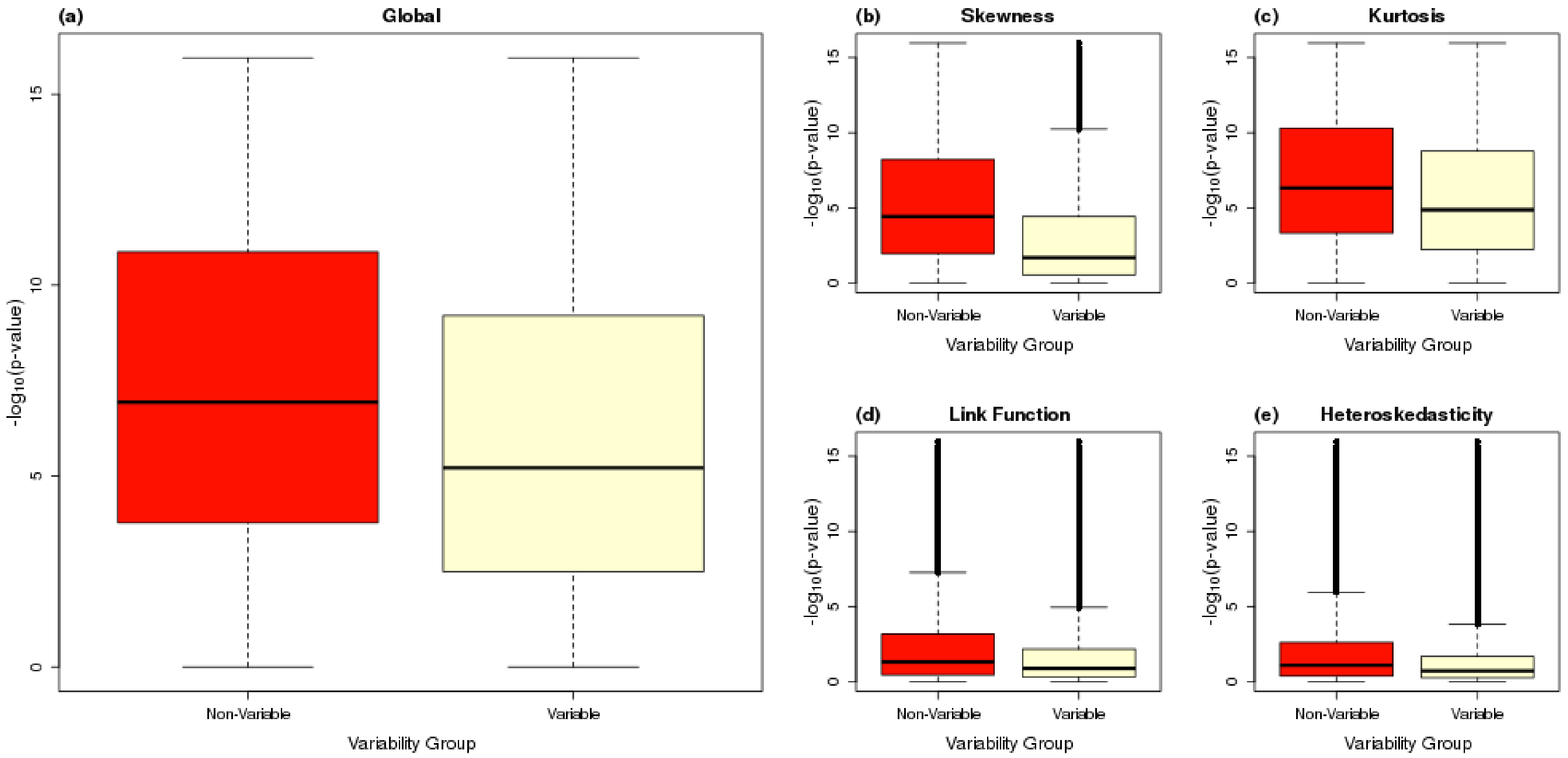

Figure S6: Boxplots of $-\log_{10}$(p-value) for each of the 5 tests: **(a)** global **(b)** skewness **(c)** kurtosis **(d)** link function and **(e)** heteroscedasticity separated by site variability status. DNA methylation sites were defined as variable if the range of their middle 80% of values, calculated as the 90th percentile (P_{90}) minus the 10th percentile (P_{10}) was greater than 5%. Each boxplot is colored by their mean $-\log_{10}$(p-value), from light yellow (lowest $-\log_{10}$(p-value)) to red (highest $-\log_{10}$(p-value). Sites with a p-value of 0 (i.e. $p < 2.22\text{x}10^{-16}$) were removed from these plots.

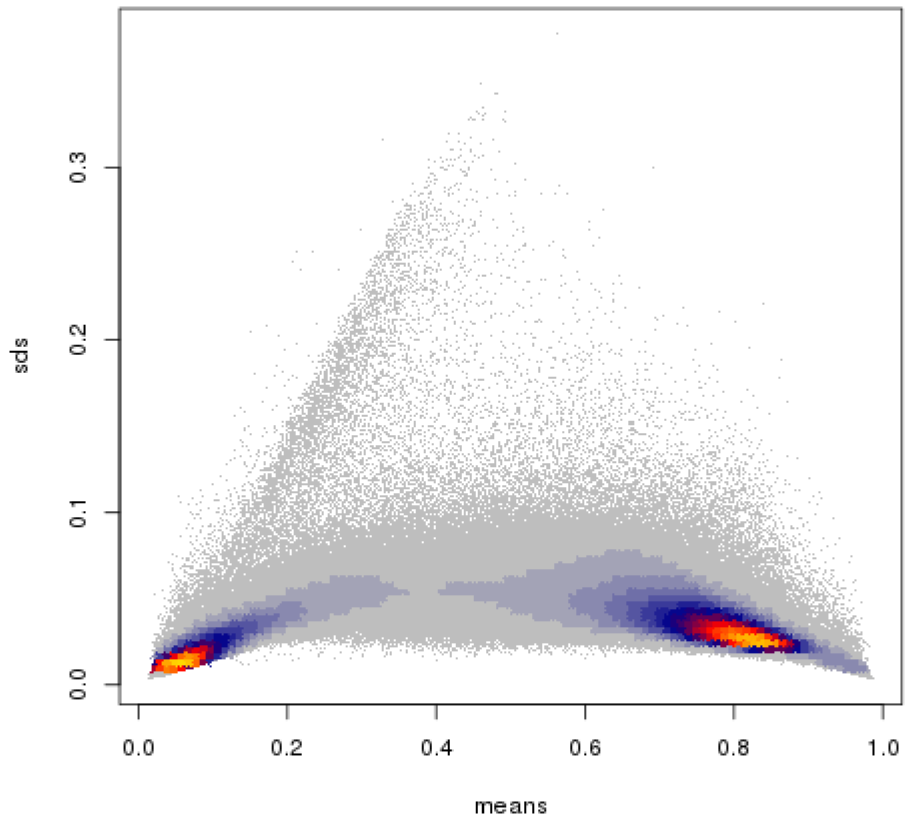

Figure S7: Scatterplot of variability (standard deviation; y-axis) against mean methylation level (x-axis), for all DNA methylation sites tested. The color of the points represents the density of points plotted (low density in grey to high density in yellow).

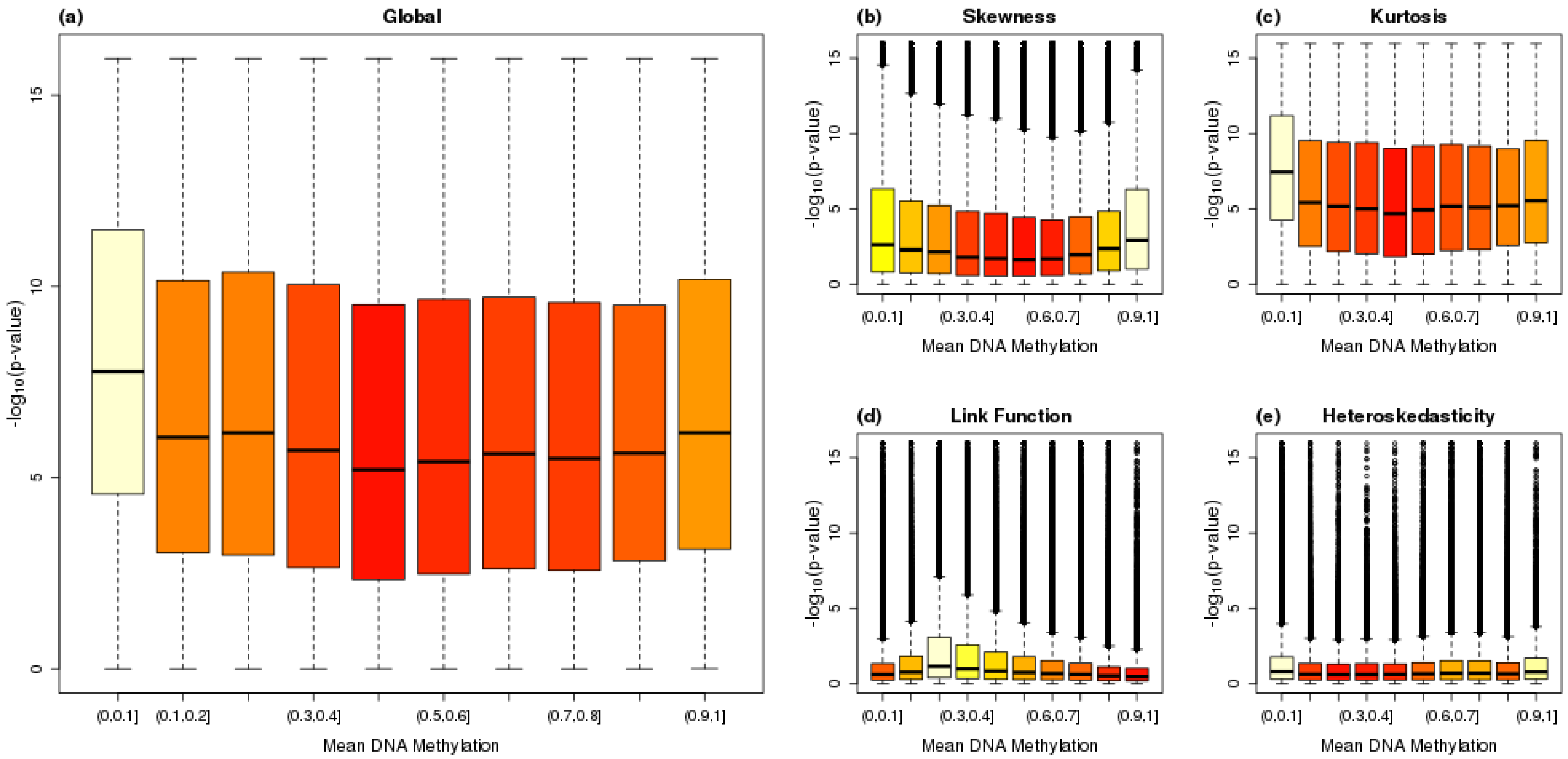

Figure S8. Comparison of suitability of linear regression assumptions for M-values across the distribution of DNA methylation levels. Boxplots of –$\log_{10}$(p-value) for each of the 5 tests **(a)** global **(b)** skewness **(c)** kurtosis **(d)** link function and **(e)** heteroscedasticity for groups of DNA methylation sites binned by their mean DNA methylation level, measured as a beta-value. The boxes are coloured by their mean –$\log_{10}$(p-value) from light yellow (low) to red (high).

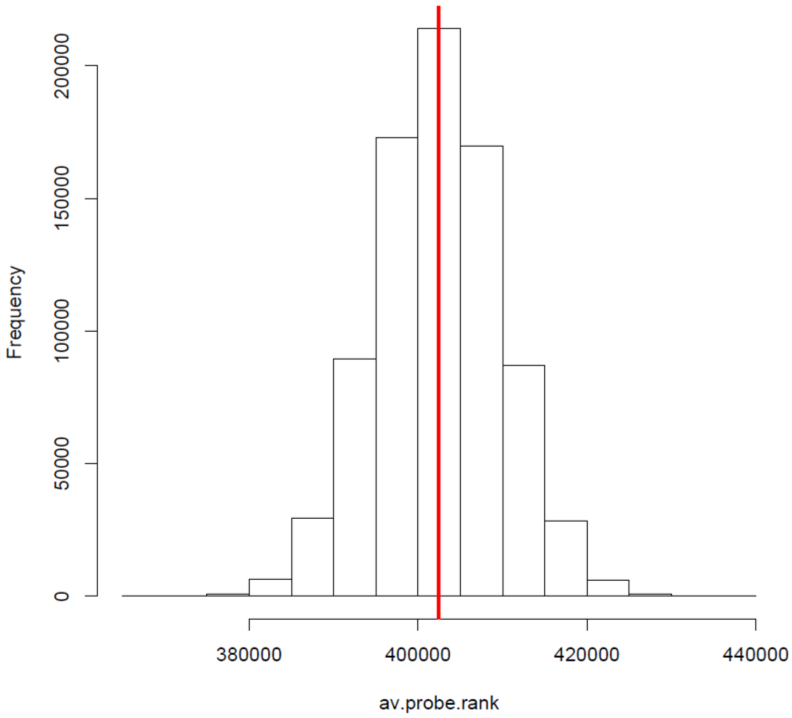

Figure S9: Histogram of DNA methylation sites mean rank from simulated null association studies. The red vertical line indicates the expected value under the scenario of no bias of 402413.5.

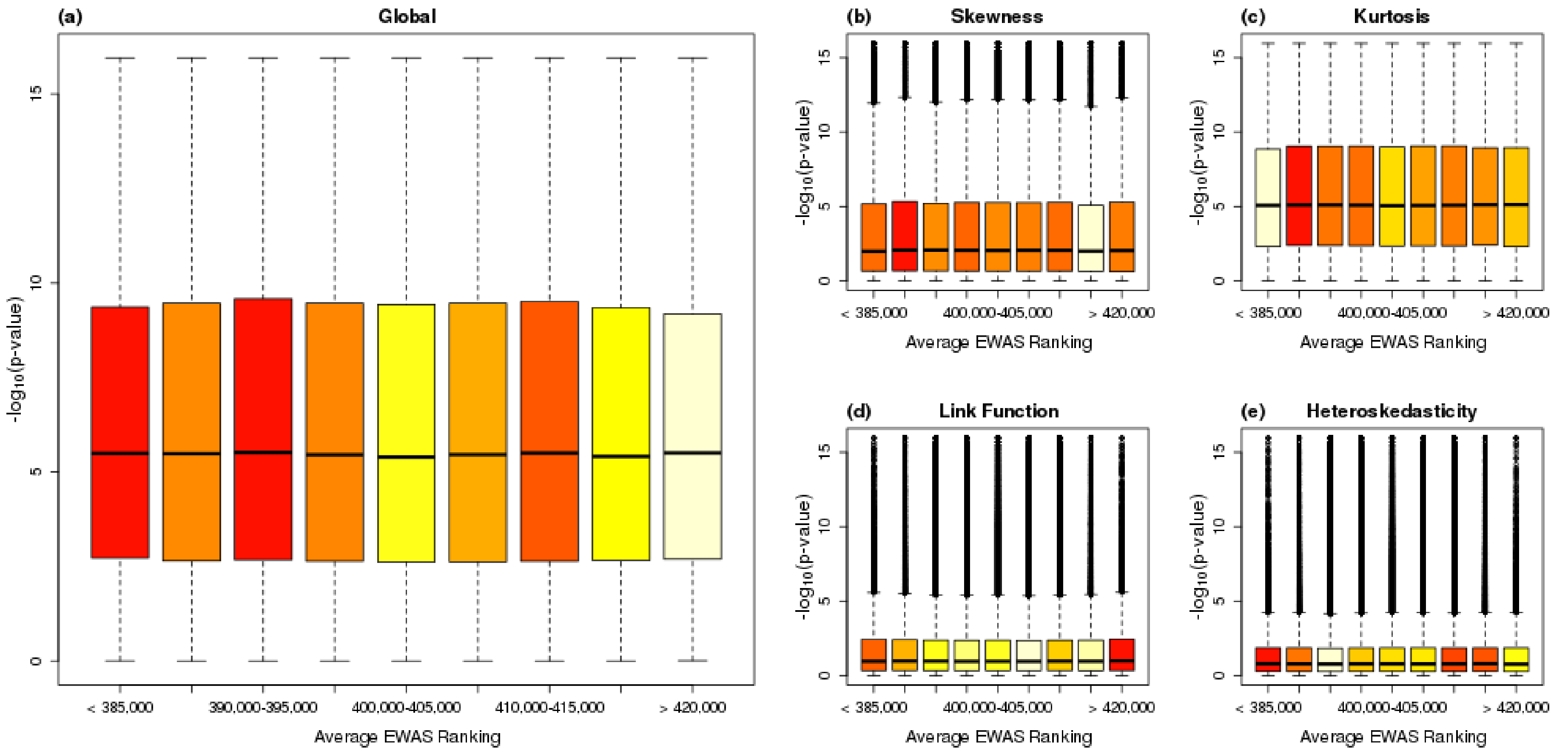

Figure S10: Boxplots of $-\log_{10}$(p-value) for each of the 5 tests: **(a)** global **(b)** skewness **(c)** kurtosis **(d)** link function and **(e)** heteroskedasticity, for groups of DNA methylation sites binned by their mean ranking from our simulations of null association studies. Sites were allocated to nine bins based on their average (mean) rank. Each boxplot is coloured based on their by their mean $-\log_{10}$(p-value) using a scale from light yellow to red in each subplot. This shows that there is generally no trend between significant p-values and mean rank. Sites with a p-value of 0 (i.e. $p < 2.22 \times 10^{-16}$) were removed for these plots.
