# Supplementary material for: Guidance for DNA methylation studies: statistical insights from the Illumina EPIC array
Source: BMC Genomics. 2019 May 14;20:366. doi: 10.1186/s12864-019-5761-7 (PMC6518823; doi:10.1186/s12864-019-5761-7)
Supplement: Supplementary file 2 — Table S1. Estimating the multiple testing correction significance threshold for sub-samples of EPIC array DNA methylation sites. Table S2. Summary of results from tests of assumptions of linear regression separated by mean DNA methylation level. The number and percentage of DNA methylation sites significant for each test (P < 9.42 × 10^–8^) split by mean DNA methylation level. Table S3. Summary of results from tests of assumptions of linear regression separated by DNA methylation standard deviation. The number and percentage of DNA methylation sites significant for each test (P < 9.42 × 10^–8^) split by DNA methylation level standard deviation. Table S4. Summary of results from tests of assumptions of linear regression separated by DNA methylation variability status. The number and percentage of DNA methylation sites significant for each test (P < 9.42 × 10^–8^). Variable DNA methylation sites are defined as those with the range of their middle 80% of values greater than 5%. Table S5. Summary of DNA methylation sites significantly rejecting the assumptions of linear regression comparing beta-values and M-values. For each of the 5 tests performed by the gvlma package the number and percentage of DNA methylation sites with significant p-values (P < 9.42 × 10^–8^) are reported for linear regression models based on beta-values and M-values. Table S6. Summary of results from tests of assumptions of linear regression separated by mean rank in null association studies. The number and percentage of DNA methylation sites significant for each test (P < 9.42 × 10^–8^) split by their mean rank across 1000 simulated null association studies. (PDF 153 kb) [file 12864_2019_5761_MOESM2_ESM.pdf]

Additional File 2: Supplementary tables accompanying "Guidance for DNA methylation studies: Statistical insights from the EPIC array"

Density	Number of sites	Mean significance threshold (P_T)		Effective number of independent tests		% of total
		Estimate	95% CI	Estimate	95% CI	
0.05	40241	1.52E-06	1.14e-06 - 1.91e-06	32800	43700 - 26200	81.6
0.15	120724	5.66E-07	4.76e-07 - 6.89e-07	88300	105000 - 72500	73.1
0.25	201206	3.68E-07	3.07e-07 - 4.44e-07	136000	163000 - 113000	67.6
0.35	281689	2.64E-07	2.11e-07 - 3.27e-07	190000	237000 - 153000	67.3
0.45	362172	2.04E-07	1.67e-07 - 2.36e-07	245000	299000 - 212000	67.7
0.55	442654	1.60E-07	1.34e-07 - 1.93e-07	313000	373000 - 259000	70.8
0.65	523137	1.37E-07	1.2e-07 - 1.59e-07	365000	417000 - 315000	69.7
0.75	603620	1.19E-07	1.05e-07 - 1.37e-07	419000	477000 - 365000	69.4
0.85	684102	1.07E-07	1e-07 - 1.19e-07	465000	500000 - 419000	68.0
0.95	764585	1.00E-07	9.42e-08 - 1.04e-07	500000	531000 - 481000	65.4
1	804826	9.42E-08	-	531000	-	65.9

Table S1: Estimating the multiple testing correction significance threshold for sub-samples of EPIC array DNA methylation sites.

Mean DNA methylation level	DNA methylation sites		Global		Skewness		Kurtosis		Link Function		Heteroskedasticity	
	N	%	N	%	N	%	N	%	N	%	N	%
0-0.1	153026	19.0	140538	91.8	102603	67.0	134974	88.2	21618	14.1	13321	8.7
0.1-0.2	58929	7.3	51533	87.4	33747	57.3	48311	82.0	8319	14.1	2309	3.9
0.2-0.3	34363	4.3	25937	75.5	12902	37.5	23682	68.9	3389	9.9	330	1.0
0.3-0.4	27842	3.5	18464	66.3	7123	25.6	16849	60.5	1712	6.1	114	0.4
0.4-0.5	29312	3.6	17363	59.2	5796	19.8	15775	53.8	1096	3.7	97	0.3
0.5-0.6	45183	5.6	27337	60.5	12338	27.3	24870	55.0	1188	2.6	235	0.5
0.6-0.7	89173	11.1	59323	66.5	31454	35.3	54674	61.3	2814	3.2	1700	1.9
0.7-0.8	186065	23.1	122718	66.0	66303	35.6	116653	62.7	8197	4.4	7260	3.9
0.8-0.9	162825	20.2	99797	61.3	49978	30.7	95365	58.6	9459	5.8	8306	5.1
0.9-1	18108	2.2	14909	82.3	10213	56.4	13307	73.5	1780	9.8	1329	7.3

Table S2: Summary of results from tests of assumptions of linear regression separated by mean DNA methylation level. The number and percentage of DNA methylation sites significant for each test ($P < 9.42x10^{-8}$) split by mean DNA methylation level.

Standard Deviation	DNA methylation sites		Global		Skewness		Kurtosis		Link Function		Heteroskedasticity	
	**N**	**%**	**N**	**%**	**N**	**%**	**N**	**%**	**N**	**%**	**N**	**%**
0-0.01	44157	5.5	37445	84.8	25897	58.6	34706	78.6	1320	3.0	2830	6.4
0.01-0.02	114154	14.2	95415	83.6	64157	56.2	91346	80.0	12550	11.0	7499	6.6
0.02-0.03	209019	26.0	130263	62.3	68476	32.8	124946	59.8	16868	8.1	9095	4.4
0.03-0.04	167646	20.8	115669	69.0	66303	39.5	111153	66.3	10985	6.6	7269	4.3
0.04-0.05	109852	13.6	80209	73.0	45371	41.3	76206	69.4	5820	5.3	3875	3.5
0.05-0.06	69563	8.6	52674	75.7	27818	40.0	49007	70.4	3548	5.1	2349	3.4
0.06-0.07	40991	5.1	30832	75.2	15077	36.8	27851	67.9	2007	4.9	1219	3.0
0.07-0.08	22323	2.8	16017	71.8	7823	35.0	13872	62.1	1183	5.3	547	2.5
0.08-0.09	11042	1.4	7323	66.3	3914	35.4	5959	54.0	680	6.2	158	1.4
0.09-0.1	4952	0.6	3200	64.6	2079	42.0	2425	49.0	535	10.8	55	1.1
> 0.1	11127	1.4	8872	79.7	5542	49.8	6989	62.8	4076	36.6	105	0.9

Table S3: Summary of results from tests of assumptions of linear regression separated by DNA methylation standard deviation. The number and percentage of DNA methylation sites significant for each test ($P < 9.42x10^{-8}$) split by DNA methylation level standard deviation.

	DNA methylation sites		Global		Skewness		Kurtosis		Link Function		Heteroskedasticity	
	**N**	**(% of all)**	**N**	**(% of group)**	**N**	**(% of group)**	**N**	**(% of group)**	**N**	**(% of group)**	**N**	**(% of group)**
**Non-variable**	174174	21.6	148395	85.2	103502	59.4	141508	81.2	19761	11.3	14441	8.3
**Variable**	630652	78.3	429524	68.1	228955	36.3	402952	63.9	39811	6.3	20560	3.3

Table S4: Summary of results from tests of assumptions of linear regression separated by DNA methylation variability status. The number and percentage of DNA methylation sites significant for each test ($P < 9.42 \times 10^{-8}$). Variable DNA methylation sites are defined as those with the range of their middle 80% of values greater than 5%.

		Global	Skewness	Kurtosis	Link Function	Heteroskedasticity
**Beta-values**	**N reject null hypothesis**	577919	332457	544460	59572	35001
	**% of all probes**	71.8%	41.3%	67.6%	7.40%	4.35%
**M-values**	**N reject null hypothesis**	569146	233776	536916	22268	12864
	**% of all probes**	70.7%	29.0%	66.7%	2.77%	1.60%
**Difference**	**N reject null hypothesis**	8773	98681	7544	37304	22137
	**% of all probes**	1.09%	12.3%	0.94%	4.64%	2.75%
**Common**	**N reject null hypothesis**	489164	159399	469567	12797	10002
	**% of significant sites using beta-values**	84.6%	47.9%	86.2%	21.5%	28.6%

Table S5: Summary of DNA methylation sites significantly rejecting the assumptions of linear regression comparing beta-values and M-values. For each of the 5 tests performed by the *gvlma* package the number and percentage of DNA methylation sites with significant p-values ($P < 9.42x10^{-8}$) are reported for linear regression models based on beta-values and M-values.

Mean rank	DNA methylation sites		Global		Skewness		Kurtosis		Link Function		Heteroskedasticity	
	**N**	**%**	**N**	**%**	**N**	**%**	**N**	**%**	**N**	**%**	**N**	**%**
< 385,000	7271	0.9	5216	71.7	3028	41.6	4939	67.9	529	7.3	312	4.3
385,000-390,000	29330	3.6	21067	71.8	11937	40.7	19766	67.4	2179	7.4	1321	4.5
390,000-395,000	89558	11.1	64433	71.9	37005	41.3	60660	67.7	6570	7.3	3792	4.2
395,000-400,000	172799	21.5	124260	71.9	71301	41.3	117021	67.7	12765	7.4	7484	4.3
400,000-405,000	214069	26.6	153735	71.8	88684	41.4	144747	67.6	15920	7.4	9324	4.4
405,000-410,000	169644	21.1	121709	71.7	70103	41.3	114763	67.6	12623	7.4	7460	4.4
410,000-415,000	87185	10.8	62477	71.7	35986	41.3	58917	67.6	6449	7.4	3748	4.3
415,000-420,000	28272	3.5	20231	71.6	11594	41.0	19108	67.6	2043	7.2	1245	4.4
> 420,000	6698	0.8	4791	71.5	2819	42.1	4539	67.8	494	7.4	315	4.7

Table S6: Summary of results from tests of assumptions of linear regression separated by mean rank in null association studies. The number and percentage of DNA methylation sites significant for each test ($P < 9.42 \times 10^{-8}$) split by their mean rank across 1000 simulated null association studies.
